# The impact of metastability on the high-pressure behavior of cerium

**DOI:** 10.1038/s41467-026-74329-w

**Published:** 2026-07-13

**Authors:** Christopher J. Ridley, Alice I. Smith, Luke L. Daemen, Bianca Haberl

**Affiliations:** 1 Neutron Scattering Division, Neutron Sciences Directorate, Oak Ridge National Laboratory, Oak Ridge, TN USA; 2 Materials Science and Technology Division, Los Alamos National Laboratory, Los Alamos, NM USA; 3 University of California, Riverside, Riverside, CA USA; 4 Department of Materials Physics, Research School of Physics, The Australian National University, Canberra, ACT Australia

**Keywords:** Structure of solids and liquids, Metals and alloys, Structure of solids and liquids

## Abstract

The structures adopted by solids under pressure are often assumed to reflect thermodynamic equilibrium, yet in many materials phase selection is strongly influenced by kinetic pathways and microstructural inheritance. Elemental cerium (Ce) exemplifies this challenge, with decades of conflicting reports describing different high-pressure crystal structures emerging under nominally identical conditions. Here we use neutron diffraction from large ( ~ 60 mm^3^) sample volumes to follow the structural evolution in ultra-high-purity Ce during controlled pressure-temperature cycling between 85 and 295 K and up to 8 GPa. We find that the crystal structure formed at high pressure depends on the compression pathway: slow compression ( ~ 0.25 GPa hr^−1^) at room temperature favors an orthorhombic phase (${\alpha }^{{\prime} }$), whereas slow ( ~ 0.25 GPa hr^−1^) and also moderately faster ( ~ 0.5 GPa hr^−1^) compression at low temperature stabilizes a pure monoclinic phase (*α*^*″*^). The low-temperature phase persists metastably over a wide temperature range but transforms irreversibly upon heating above  ~ 280 K, or modest pressure cycling. Remarkably, the lower-pressure *γ* phase remains trapped far beyond the expected stability field, persisting to the highest pressures studied. These observations show that phase selection in Ce is governed by kinetics and microstructural memory rather than equilibrium thermodynamics or sample morphology alone, establishing path dependence as a defining feature of its high-pressure behavior.

## Abstract (web summary)

Using neutron diffraction, the authors show that cerium’s crystal structure under pressure depends on its pressure–temperature path, with metastable phases and microstructural memory controlling which high-pressure phase forms.

## Introduction

Solid-solid phase transitions under pressure are often interpreted in terms of equilibrium phase diagrams, yet in many materials, the structure that forms depends strongly on kinetic pathways, metastability, and retained microstructure^1–4^. Diffusionless transformations can preserve crystallographic orientation and phase fractions far beyond their expected stability fields^5^, leading to pronounced hysteresis and path dependence^6–8^. The rare-earth metal cerium (Ce) exemplifies these effects: at pressures of 4–6 GPa, nominally identical compression conditions have been reported to produce different high-pressure crystal structures^9^, with sample morphology identified as a method to control the transformation pathway^10–12^. Despite extensive study, the mechanisms governing the selection between these competing phases, particularly the roles of kinetics, metastability, and microstructural inheritance, remain unresolved, especially at low temperature.

Ce exhibits exceptionally rich structural and electronic complexity, with four ambient-pressure polymorphs^13^, a minimum in its melt curve^14^, a pressure-induced electronic transition^15–17^, and the rare occurrence of a solid-solid phase boundary terminating at a critical point^18,19^. These behaviors originate from the dual localized-itinerant character of the single 4*f* electron^20^, which drives strong coupling between electronic and elastic degrees of freedom and poses a longstanding challenge for theory and experiment^21–25^. At ambient conditions, Ce crystallizes in the face-centered cubic *γ* phase ($Fm\overline{3}m$)^26^, transforming on cooling to the hexagonal *β* phase (*P*6_3_/*mmc*)^27^ and subsequently to the isostructural face-centered cubic *α* phase below ~100 K^15^, while heating above 1000 K yields the body-centered cubic δ phase ($Im\overline{3}m$)^28^. Under compression at room temperature, Ce undergoes a first-order *γ* → *α* transition above ~0.8 GPa with a 15% volume collapse, terminating at a critical point near ~1.5 GPa and 480 K^19,29^. Further compression to ~4 GPa stabilizes either the orthorhombic ${\alpha }^{{\prime} }$ (*Cmcm*) or monoclinic *α*^″^ (*C*2/*m*) phase^16,30,31^, followed by formation of the body-centered tetragonal *ϵ* phase (*I*4/*mmm*) near 12 GPa, which persists to at least 208 GPa^32,33^.

Heating above ~400 K irreversibly eliminates the monoclinic *α*^″^ phase, yielding pure orthorhombic ${\alpha }^{{\prime} }$^11,34,35^, while further heating induces reversible transitions to either the *α* or *ϵ* phases depending on pressure^11,34,35^. Consistently, in situ compression at elevated temperature produces direct nucleation of ${\alpha }^{{\prime} }$ from *α* at 4 to 6 GPa^36^. At lower temperatures, however, reported phase boundaries vary widely: some phase diagrams show coexistence of ${\alpha }^{{\prime} }$ and *α*^″^ below ~400 K^11,34,37^, while others report only *α*^″^^13^, which has itself been described as metastable^35^. Low-temperature compression studies are scarce, with most experiments relying on room-temperature compression followed by cooling^12,38^, and only one low- temperature isotherm reported^39^. As a result, the temperature dependence of ${\alpha }^{{\prime} }$ versus *α*^″^ formation, their relative stability, and the mechanisms governing their nucleation remain poorly understood.

Here, we use high-pressure neutron diffraction in a large-volume Paris–Edinburgh (PE) press to investigate the phase behavior of ultra-high-purity Ce up to ~8 GPa. By combining room-temperature and low-temperature isotherms with controlled pressure and temperature cycling above 4 GPa, we directly probe phase coexistence, metastability, and transformation pathways. This approach enables new insight into the kinetic origin of ${\alpha }^{{\prime} }$ vs. *α*^″^ phase selection in Ce and clarifies the role of pathway dependence and retained microstructure in shaping its high-pressure phase diagram.

## Results and discussion

### Compression at 295 K

**Fig. 1:**
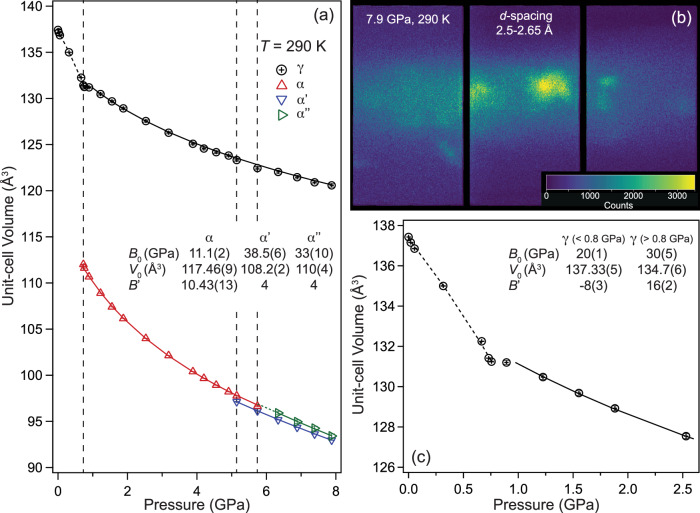
Lattice behaviour of Ce at 290 K. **a** Unit-cell volume and phase evolution of four phases of Ce versus pressure at 290 K (slow compression). Here, *γ* is seen to coexist up to the maximum pressure considered, with a clear change in compression at the transition to *α*. At 5.14(7) GPa, the sample begins to transition to ${\alpha }^{{\prime} }$, at 5.74(8) GPa, *α*^″^ is also observed. By 6.34(9) GPa, *α* has disappeared, and a mixture of $\gamma /{\alpha }^{{\prime} }/{\alpha }^{{\prime\prime} }$ remains. Solid lines are fits to Rydberg–Vinet equations of state, the parameters for which are shown in each case, treating *γ* differently above and below 0.8 GPa. **b** Two-dimensional view of a subset of detector modules (SNAP, ORNL), filtered to show a narrow *d*-spacing range (2.5–2.65 Å), collected at 7.9(2) GPa, showing the high degree of texture apparent in ${\alpha }^{{\prime} }$. **c** Unit-cell volume of *γ* in the low-pressure region, showing the softening (${B}^{{\prime} } < 0$) leading up to the transition to *α*, and the subsequent anomaly in the unit-cell volume, showing almost no change (or a slight increase within error) while *α* decreases continuously. The central values and errors were extracted from the Rietveld refinements. The error bars are obscured by the symbols in some instances. Source data are provided as a Source data file.

At close to ambient conditions, the first pellet of Ce was found to be a mixed phase of *γ* and *β*, consistent with Lawson and Tang^15^. In Fig. 1a, we observe ‘softening’ of the *γ*-phase as a prelude to the transition to *α*, consistent with piezometry^18,40,41^ and previous neutron diffraction measurements^17^. By 0.730(6) GPa, *β* disappears, and *α* grows in. Notably, while the phase fraction of *γ* reduces significantly with further pressure, it persists up to the highest pressure considered, 7.9(2) GPa. We note an anomaly in the unit-cell volume of *γ* in the low-pressure region just above the transition point ~0.8 GPa, as previously observed from X-ray diffraction measurements^19,42^, (see Fig. 1c). This was not observed by Jeong et al.^17^, likely due to the limited number of pressure points collected in that region. This anomaly may also be evident in ultrasonic measurements, with a small kink in the adiabatic bulk modulus at approximately 1.1 GPa, just into the stability region of *α*^29^. Beyond this pressure, the unit-cell volume of the metastable *γ* reduces again, though with a significantly enhanced bulk modulus (see Fig. 1a). Further comparisons of the bulk modulus with the literature values can be found in the SI Supplementary Note 1.

Anomalies in the region of the *γ* → *α* transition have been discussed extensively in the literature, such as in the behavior of the Grüneisen parameter^43^, related to the softening of the bulk modulus under pressure, and subsequent large derivative of the bulk modulus in *α*. The unit-cell volume expansion in the region of coexistence is seemingly a distinct phenomenon. The observation that the crystallinity and orientation of single-crystal samples of Ce are preserved through the *γ* → *α* transition led Decremps et al.^42^ to suggest that this anomaly could be explained by the transformation being diffusionless and shearless (unconventional martensitic-like), resulting in a large concentration of edge dislocations between the two coexisting phases. The apparently negative value of *B´* of the metastable *γ* arises from the competition between normal elastic compression and pressure-induced nucleation of the stable *α*, with the latter dominating through stress relaxation at phase boundaries rather than indicating true negative compressibility. The martensitic-like transition is supported by X-ray tomography measurements, showing that platelet-shaped crystallites of *α* nucleate within *γ* with the same orientation^44^. Elastic softening and anisotropy drive a diffusionless, free-energy-minimizing phase transformation, forming platelet microstructures, while edge dislocations at phase interfaces accommodate the large lattice mismatch, preserving the crystal orientation. Our neutron diffraction measurements are in agreement with this, showing a clear coexistence of *γ* through a wide stability field. The high penetration depth of neutrons provides unparalleled sensitivity to bulk phase-fraction quantification, revealing that ~4(1) wt% *γ*-Ce persists beyond the stability field of *α* and exhibits a pronounced change in compressibility. The possibility that this is instead one of the typical impurity phases, namely CeO, CeO_2_, or CeH_2_, emergent at high pressures, or PbO from the pressure marker, is discussed more extensively in the SI Supplementary Note 2, and ultimately eliminated.

**Fig. 2:**
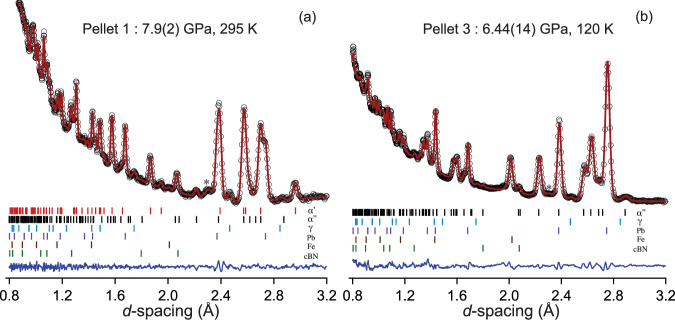
Fits to neutron powder data. Rietveld fits to neutron powder diffraction data from two Ce pellets. **a** Pellet 1, at 7.9(2) GPa and 295 K. The tick marks from top to bottom index ${\alpha }^{{\prime} }$-Ce, *α*^″^-Ce, *γ*-Ce, Pb, Fe, and cBN, respectively. **b** Pellet 3, at 6.44(14) GPa and 120 K. The tick marks from top to bottom index *α*^″^-Ce, *γ*-Ce, Pb, Fe, and cBN, respectively. Note the clear absence of ${\alpha }^{{\prime} }$-Ce in the low temperature data (see deconvolved Rietveld in SI). In both plots, the observed data are represented with black markers, the calculated profile is a red line, and the residual between the observation and fit is shown as a blue line. Pb is the pressure marker, while the Fe and cBN contributions come from the anvils used in the Paris–Edinburgh press. The peak marked (*) at a *d*-spacing of 2.31 Å is a background peak from the press itself, observed in the empty press measurement, and with all pellets. It doesn’t shift with pressure or change in intensity. For both figures, the data were accumulated from a single measurement collected over a period of approximately 1 h. The error bars represent counting statistics on these data and are shown, though are smaller than the data symbols used. Source data are provided as a Source data file.

The unit-cell volume of the *α* phase shows more conventional behavior with pressure. At approximately 5.14(7) GPa, the *α* phase fraction starts to reduce, giving way to ${\alpha }^{{\prime} }$. At the next pressure step, 5.74(8) GPa, a small trace of *α*^″^ begins to emerge, and by 6.34(9) GPa, *α* is no longer present in the pattern. From this pressure up to 7.9(2) GPa, ${\alpha }^{{\prime} }$, a small amount of *α*^″^, and *γ* remain. The transition pressure is approximately 1 GPa higher than reported by Schiwek et al.^37^, though a similar discrepancy in transition pressure was also reported by Munro et al.^11^, and is in general agreement with previous measurements^45,46^. As observed in other measurements of ${\alpha }^{{\prime} }$, the diffracted signal is highly textured, approaching a single-crystal-like level of preferred orientation observed on the 2D image of the detector^10,11,45,47^ (see Fig. 1b). The sample is illuminated with a white beam of neutrons, with each energy distinguishable through the differing time-of-flight from the source to the detector. Figure 1b is filtered to show only the neutron signal over the 2.5–2.65 Å *d*-spacing range, represented over the entire detector face, showing single-crystal-like features, representing the strong texture in the (002) and (021) reflections of ${\alpha }^{{\prime} }$, the only reflections present in this *d*-spacing range. The persistence of the trace *γ* phase through the transition is curious, remaining at the ~4(1) wt% level while *α* fully transforms, leaving a mixture of 79(1) wt% ${\alpha }^{{\prime} }$, 6.6(7) wt% *α*^″^, with the balance being made up by Pb and anvil contributions (see Fig. 2a). This suggests that a microstructure effect, akin to the *γ* → *α* transition, may be active, partially explaining the strong texture observed in the orthorhombic phase, both here and in many other studies^10,33,47^. Gu et al.^45^ found that ${\alpha }^{{\prime} }$ tends to recrystallize from *α*-centers.

It was recently shown that a critical grain size influences the *α* → *α*^″^ transition^48^, with coarse grains (produced through annealing the starting material between 670 and 870 K) promoting ${\alpha }^{{\prime} }$, and fine grains favoring *α*^″^^49^. In the present samples, the starting point was a pre-pressed pellet of coarse Ce powder and Pb foil, such that particle size, cold-work defect density, residual strain, and texture are inherently coupled. The observed ${\alpha }^{{\prime} }$/*α*^″^ selection is therefore attributed to the retained microstructural state rather than grain size alone. Pb was used as an in situ pressure marker; while a secondary mechanical role in redistributing local stresses cannot be excluded, the strong dependence of phase selection on pressure–temperature pathway across identically prepared pellets indicates that retained Ce microstructure, rather than Pb content itself, is the dominant factor. Following the work of Henry et al.^44^, it is therefore extremely likely that the amount of remnant *γ* phase present affects this process.

McMahon and Nelmes ^9,10^ recognized that samples of Ce prepared via cutting appear to yield a majority ${\alpha }^{{\prime} }$, whereas those made from filings, or those cold-worked into thin plates, yield a majority *α*^″^ (see SI Table S3). Our measurements expand on this finding. Using toroidal anvils with their cupped recession for sample space allows many magnitudes of order larger sample volume, coupled with potentially lower levels of deviatoric stresses (see SI Supplementary Note 3). Now, even when starting with a coarse powder, akin to filings, subsequently cold-worked into a dense pellet prior to further pressurization, a majority ${\alpha }^{{\prime} }$ forms with a trace of *α*^″^, an outcome previously only observed from “cut-pieces” (100–200 μm across). In this context, diffraction studies probing only small sample volumes may misrepresent phase fractions in a strain-heterogeneous material, whereas large-volume neutron diffraction provides a more representative measure of the phase coexistence. It has been noted by Ma et al.^47^ that ${\alpha }^{{\prime} }$ is only reported in studies where the compression of the sample is slow, as in the present study, and that pre-compression of Ce-slices (held at ~1.4 GPa for ~72 h) leads to a growth of nucleation centres for ${\alpha }^{{\prime} }$ formation. This presumably forms coarser grains, making the theory consistent with Lai et al.^49^. In the present study, the precompression (during formation of the pellet) was ~0.6 GPa, held for no longer than 1 min. Zachariasen and Ellinger^31^ observed a mixture of ${\alpha }^{{\prime} }$ and *α*^″^ consistently, with no measurable change in a sample held at pressure for 10 days, but pressure cycling was shown to encourage the formation of ${\alpha }^{{\prime} }$. Indeed, it is striking how similar in volume *α* and *α*^″^ are at the onset of the transition, and how small the volume reduction to ${\alpha }^{{\prime} }$ is (see Fig. 1a).

### Low temperature compression

A second pellet of Ce was cooled at ambient pressure to 100 K over approximately 1 h. The resulting sample was found to be a mixture of 77(2) wt% *γ* & 23(2) wt% *β*. Compression to 0.074(6) GPa, and the sample becomes mixed *γ*/*β*/*α*. Further compression to 0.268(7) GPa shows the unit-cell volumes extracted for the *γ* and *β* phases exhibit a slight increase with increasing pressure, in contrast to the volume reduction observed for the emergent *α* phase. This anomaly is far more pronounced than that observed in the room temperature compression. On close inspection of the Rietveld fits over this pressure region, there is a strong *hkl*-dependent peak offset, which could be indicative of non-hydrostatic conditions (see SI Fig. S4). It is understood that deviatoric stresses can develop under high-pressure conditions without a pressure medium present, which can be exacerbated at low temperatures. The pressure assembly used here, consisting of toroidal anvils with cupped recesses for the sample, typically generates low levels of deviatoric stresses, as demonstrated at room temperature using a test sample of NaCl^50^, which has a similar bulk/shear modulus to Ce. The scattering geometry used in the present study uses only a narrow plane through the center of the sample/gasket cross-section, where the lowest levels of deviatoric stresses were observed^50^ (discussed further in the SI). While the potential effects of these stresses cannot be dismissed outright, we suggest that our observation is more likely related to the metastability of *γ* at this temperature, kinetically trapped through fast cooling. Coupled with the effects of *α* nucleation, as interpreted by Decremps et al.^42^, this explains the apparent volume increase observed over the low-pressure mixed phase region. This *hkl*-dependent behaviour does not continue in the isostructural *α* phase (see SI Fig. S4). At a constant applied load, the sample was then heated to 190 K, removing all traces of *β*, and cooled back to 100 K, yielding a mixture of *α* with a small trace of *γ*. Thereafter, compression continued at a constant 100 K.

Above 5.12(6) GPa, additional peaks appear in the pattern which can only be fully accounted for by monoclinic *α*^″^. The lack of ${\alpha }^{{\prime} }$ is very clear, the (112) reflection is absent, and there are several reflections (e.g., at 1.6, 2.07, 2.24, and 2.64 Å in *d*-spacing) which are unaccounted for by the orthorhombic cell. This is in agreement with predictions from computational studies that *α*^″^ is the only stable phase at low temperatures^51,52^, a symmetry not considered by earlier some work based on the opposite assumption^53^, and consistent with the phase evolution at 93 K reported by Olsen et al.^39^. Above 2.75(2) GPa, *γ* remains at an almost undetectable trace level. By 6.32(9) GPa, there was no longer any trace of *α* remaining. At ~6.31(9) GPa, the sample was temperature cycled; warmed slowly from 85 to 285 K over the course of approximately 12 h, collecting data at 40 K intervals. During this process, the pressure drifted up to 8.4(2) GPa, but the sample remained *α*^″^. The sample was subsequently cooled back to 85 K over 30 min, at which point a very small trace of the (112) reflection of ${\alpha }^{{\prime} }$ started to emerge at a *d*-spacing of 1.87 Å. The sample remained at base temperature for 30 min before being heated quickly back to 295 K over 2 h. After this temperature cycle, the sample formed more clearly into ${\alpha }^{{\prime} }$. This observation is consistent with the findings of Zhao and Holzapfel^34^ that *α*^″^ appears metastable, with ${\alpha }^{{\prime} }$ forming irreversibly on heating, though we believe this to be the first direct structural evidence of metastability down to 100 K. It is also possible that the rate of temperature cycling was not the determining factor, rather the maximum temperature it was taken to (285 K vs. 295 K). On pressure release at 290 K, the sample reverted back to *α* and then *γ* as expected.

In a third pellet, the starting Ce was initially single-phase *γ*. Compression at room temperature to 1.126(5) GPa over approximately 45 min, faster than the first loading (~12 h), resulted in transformation to the *α* phase with a trace of *γ*. The sample was then cooled to 120 K and further compressed. Above 5.55(7) GPa, the monoclinic *α*^″^ phase nucleated and was fully established by 6.44(14) GPa (Fig. 2b). The structure remained monoclinic upon subsequent heating to 200 K at 7.52(3) GPa and further to 285 K at 8.25(4) GPa. At 285 K, partial decompression to 7.34(2) GPa induced the appearance of a weak ${\alpha }^{{\prime} }$ signature, which persisted upon re-compression to 7.86(5) GPa. This demonstrates that pure *α*^″^ can be transformed into orthorhombic ${\alpha }^{{\prime} }$ through modest pressure cycling, consistent with earlier observations by Zachariasen and Ellinger^31^ and with the small unit-cell volume difference between the two phases (Fig. 1a). By contrast, McMahon and Nelmes^9^ reported the reverse transformation at room temperature, with ${\alpha }^{{\prime} }$ converting to *α*^″^ via decompression into *α* followed by re-compression. The apparent discrepancy arises from whether the transformation pathway allows the system to revert to the *α* phase. Our results show that pressure cycling within the *α*^″^ stability field, without passing through *α*, instead favors the formation of ${\alpha }^{{\prime} }$, the denser and less distorted structure. This behavior supports a central role for the transformation pathway and retained microstructure in governing phase selection.

### Considering the influence of sample purity

In the present study, particular attention was paid to purifying the starting Ce metal (see “Methods,” and SI Table S2 for a summary of other studies). In particular, Ce has a high affinity for H, which diffuses through the bulk of the material, as observed in spectrographic analysis^54^. Removing this is important to avoid the formation of any stable hydride phases (CeH_2_) under high pressure^55^, which has been observed in many high-pressure studies^9,47^, and later recognized in others^45,56^. There is little explicit mention of the effects of purity on the high-pressure phase diagram in the literature, though the findings of McMahon and Nelmes^9^ suggest that both 99.9 and 99.99% (on a metals basis) show the same effects, and speculate that the absence or presence of a CeH_2_ impurity has no effect. Chesnut et al.^57^ observed that the unit-cell volume of the starting *γ* phase and the sharpness of the $\alpha \to {\alpha }^{{\prime} }$ transition depended on sample purity, although both high- and lower-purity samples ultimately transformed to ${\alpha }^{{\prime} }$. Gschneidner Jr et al.^54^ were able to form an intermediate cubic-phase, isostructural to *γ* with a slightly reduced unit-cell volume, by quenching *γ* in liquid-H_2_, and then heating. Zhao and Holzapfel^34^ were careful to assay for H-content and observed no hydride. Tsiok and Khvostantsev^35^ observed that Ce absorbed a large amount of H, resulting in very different P/T behavior with the appearance of unsolved new phases. Ultimately, the effect of low-level H (or other) impurities on the phase behavior of metals under compression is not well studied, while for semiconductors, such as (amorphous) Si and Ge, volatile impurities are well known to dramatically alter the expected phase behavior^58,59^. For Ce, the known effects of grain-size on the phase behavior suggest that volatile impurities should be expected to alter the phase behavior significantly, through altering grain boundary mobility, as e.g., for Bi in Au^60^. In the present study, special care was taken to minimize H-content, both in the starting material and during loading, and no trace of hydride, oxides, or other impurities is observed.

### Towards a phase diagram

**Fig. 3:**
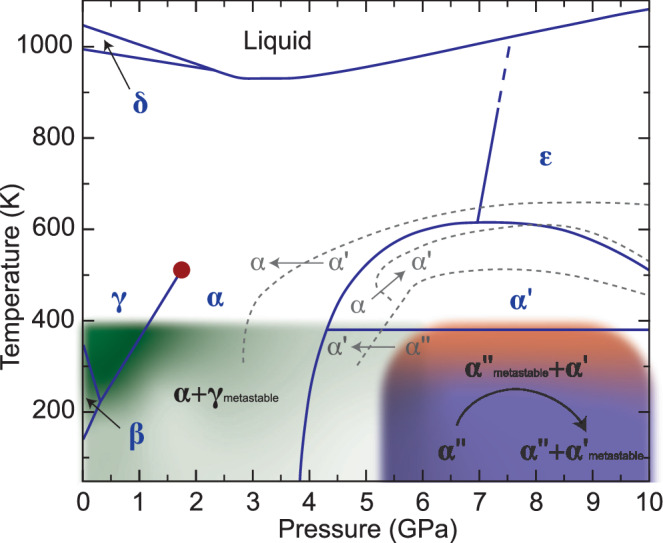
Updated phase diagram of Ce. Phase diagram of Ce adapted from Schiwek et al.^37^ (solid blue lines), Lipp et al.^19^ (red dot), Tsiok and Khvostantsev^35^ (dashed gray lines). The (meta-)stability regions of the *γ*-phase from the current measurements are shown shaded in green. The region of stability for ${\alpha }^{{\prime} }$ and *α*^″^ from this study are shaded red to blue, respectively. It should be noted that a large hysteresis is reported for the $\alpha \to {\alpha }^{{\prime} }$ transition^13,35^. The black arrow represents temperature and pressure cycling. The space-group assignments for these phases are: *γ* and *α* ($Fm\overline{3}m$), *β* (*P*6_3_/*mmc*), δ ($Im\overline{3}m$), ${\alpha }^{{\prime} }$ (*Cmcm*), *α*^″^ (*C*2/*m*), *ϵ* (*I*4/*mmm*). Source data are provided as a Source data file.

Figure 3 places our results in the broader context of the Ce phase diagram. Our findings demonstrate that metastability is a central feature of this diagram: the *γ* phase persists metastably to at least ~8 GPa and influences the selection between the monoclinic *α*^″^ and orthorhombic ${\alpha }^{{\prime} }$ phases at higher pressure. Under low-temperature compression, identically prepared pellets consistently transform into purely monoclinic *α*^″^-Ce, with no detectable ${\alpha }^{{\prime} }$-Ce. In contrast, room-temperature compression produces a highly textured orthorhombic ${\alpha }^{{\prime} }$-Ce phase with a small coexisting fraction of *α*^″^-Ce. The low-temperature monoclinic phase shows no such texture.

While the *ϵ*-phase is included in the high-temperature region of the phase diagram following Zhao and Holzapfel^34^, its metastable character remains under discussion^35^. At room temperature, *ϵ*-Ce has been reported to form upon compression at 12–13 GPa^11,45,56^. However, on a low-temperature compression isotherm, previous X-ray studies reported its onset only at 26 GPa^39^, implying a negative slope of the relevant phase boundary. This is consistent with our observations, in which no evidence for *ϵ*-Ce was found over the pressure/temperature range investigated.

At room temperature, the two high-pressure phases discussed here, ${\alpha }^{{\prime} }$-Ce and *α*^″^-Ce, are energetically similar, such that modest variations in pressure or temperature favor formation of the denser orthorhombic structure. Consistent with this, ${\alpha }^{{\prime} }$ forms at slightly lower pressure than *α*^″^, and compression of mixed ${\alpha }^{{\prime} }$/*α*^″^ samples above 8 GPa eliminates the *α*^″^ component^45^, as does cyclic compression^31^. These observations, together with our own pressure-cycling experiments, suggest a dome-shaped region of metastability for the monoclinic *α*^″^ phase. While such behavior is qualitatively consistent with earlier studies^13,35^, the metastable regime we observe is markedly narrower and extends to lower temperatures, potentially reflecting the ultra-high purity of our samples.

Crystallographically, ${\alpha }^{{\prime} }$ and *α*^″^ are closely related through a group–subgroup symmetry relationship. The *α*^″^ (*C*2/*m*) structure can be generated from ${\alpha }^{{\prime} }$ (*Cmcm*) by a monoclinic shear of the orthorhombic cell together with a secondary displacement of the Ce atoms (ISOVIZ file attached as Supplementary Data 1). In this sense, *α*^″^ may be viewed as a symmetry-lowered distorted form of ${\alpha }^{{\prime} }$, rather than a reconstructive rearrangement. This is consistent with the small volume difference between the two phases and with the ease with which modest heating or pressure cycling seeds ${\alpha }^{{\prime} }$ and *α*^″^. Prior work likewise argued that the orthorhombic and monoclinic forms of Ce are linked by a common distortion mechanism and that their relative stability is highly sensitive to elastic fields and sample preparation^36^. This close crystallographic relationship also suggests a natural route by which retained microstructure may influence phase selection.

As the ${\alpha }^{{\prime} }\to {\alpha }^{{\prime\prime} }$ transformation requires only a small shear of the cell together with a secondary Ce displacement, defects capable of storing or accommodating local shear, such as dislocation structures, intervariant boundaries, and possibly planar faulting, may locally favor one distortion state over the other. In our measurements, the anomalous *hkl*-dependent peak shifts observed for *γ*-Ce in the low-pressure mixed-phase region at 100 K, but not in the emergent isostructural *α*-phase, together with the strong texture of ${\alpha }^{{\prime} }$ formed during slow room-temperature compression and the persistent retention of remnant *γ*-Ce, are consistent with such a defect-rich inherited microstructure. Fault-related *hkl*-dependent peak shifts and line-shape changes are well documented in other *fcc* metals^61,62^, and their prominence is known to vary strongly with deformation pathway^63^. High stacking-fault probabilities under deformation have been reported for Ce^64^, and there is some evidence for internal strain or stacking faults in Pr under high pressure^65^. In the present study, the absence of similarly strong *hkl*-dependent shifts for *γ*-Ce at room temperature likely reflects the greater ability of the defect structure to relax and redistribute stress during compression, whereas at low temperature, the inherited anisotropic microstructure is more effectively retained. In this view, the low-temperature peak shifts are consistent with frozen-in defect-mediated shear accommodation, rather than with a generic property of the *fcc* lattice itself. We therefore regard stacking faults as a plausible contributor to shear accommodation within the broader microstructural picture of Ce, although we cannot establish them as the unique mechanism selecting between ${\alpha }^{{\prime} }$ and *α*^″^.

When combined with recent reports highlighting the role of grain size in the transformation pathway^49^, these results raise a fundamental question: what is the equilibrium low-temperature phase of Ce at high pressure? Our data indicate that both ${\alpha }^{{\prime} }$ and *α*^″^ can exist as metastable states, as indicated in Fig. 3. Compression at low temperature consistently produces *α*^″^, which begins to transform into ${\alpha }^{{\prime} }$ upon heating above ~280 K; once formed, ${\alpha }^{{\prime} }$ can be kinetically trapped on subsequent cooling. Such kinetic trapping naturally favors the denser of the two structures, explaining the persistence of ${\alpha }^{{\prime} }$. These findings suggest that *α*^″^ is the high-pressure equilibrium phase at low temperature.

We show that under room-temperature compression in a large-volume cell with reasonable hydrostaticity, ultra-high-purity Ce transforms into a highly textured orthorhombic ${\alpha }^{{\prime} }$ (*α*-U) structure with a small coexisting *α*^″^ fraction. This is a surprising finding for a sample formed by cold working a coarse powder, such as ours, since previous observations on samples with similar preparation and morphology yielded pure *α*^″^ phase ^9^. We further present the first low-temperature neutron diffraction study of Ce under these conditions, demonstrating that compression at 100–120 K consistently produces the monoclinic *α*^″^ phase without coexistence of ${\alpha }^{{\prime} }$. These results establish that phase selection in Ce is strongly dependent on transformation pathway and retained microstructure, with the low-temperature *α*^″^ phase recoverable metastably to near room temperature. Gentle pressure cycling of *α*^″^ at room temperature seeds the formation of ${\alpha }^{{\prime} }$, while subsequent heating and pressure cycling allow traces of ${\alpha }^{{\prime} }$ to persist upon cooling to 85 K. Given the small volume differences between the *α*, ${\alpha }^{{\prime} }$, and *α*^″^ phases at the transition, modest thermal or pressure perturbations naturally promote gradual transformation toward the densest structure (${\alpha }^{{\prime} }$), which can then be kinetically trapped at low temperatures. Across all compression pathways, trace quantities of the lower-pressure *γ* phase persist far beyond the expected stability field.

Based on the anomalous lattice response of *γ* and recent insights into microstructural evolution^44,48^, we propose that remnant *γ* plays a key role in promoting the strongly textured formation of ${\alpha }^{{\prime} }$. Slow room-temperature compression preserves the greatest fraction of trapped *γ*, biasing the $\alpha \to {\alpha }^{{\prime} }$ transformation, whereas faster compression suppresses *γ* retention and instead favors formation of *α*^″^. Large-volume neutron diffraction is essential for revealing this persistent phase coexistence, underscoring the central roles of compression rate, thermal pathway, metastability, and microstructure in shaping the high-pressure phase diagram of Ce.

## Methods

High-pressure experiments were performed using two PE presses^66^, both equipped with single-toroidal cubic-BN anvils^67^. A VX3-type PE press was used for room temperature compression, and a VX5-type PE press was used for the two low-temperature measurements^68^. Both presses were hydraulically-driven via an automated pump. In all loadings, null-neutron-scattering TiZr gaskets^69^, with full encapsulation, allowed for a starting sample volume of approximately 60 mm^3^. For all experiments, the presses were placed in a geometry so that the beam entered and scattered through the gasket. 3D-printed B_4_C slits were used to shape the incident beam to minimize parasitic scatter^70^. Despite this, the high compressibility of Ce combined with progressive thinning of the gasket material, led to the appearance of additional diffraction contributions at higher applied loads in some of the measurements. In particular, some datasets show reflections from Fe, originating from the steel frets used to bind the anvils, as well as weak contributions from the cBN anvil material itself, predominantly in the back-scattering detector.

High-purity Ce powder was obtained from the Materials Preparation Center (Ames National Laboratory, IA, USA). The 99.999% pure starting metal was annealed under vacuum (over several days) to remove hydrogen, and vacuum cast (10^−5^ to 10^−6^ torr) to remove volatiles. The material is assayed for H content before and after annealing or recasting under vacuum in a Ta ampoule. The annealing and vacuum casting process was repeated several times. The material was sealed into glass ampoules under vacuum for transport.

For each experimental run, the Ce powder was retrieved from the glass ampule inside a He-filled glove box. A total of ~320 mg of sample material was weighed into a mortar. To act as pressure maker, several small pieces of Pb-foil (thickness ~100 μm) were added for a total of ~70 mg of Pb. This translates to a mass ratio for Ce:Pb of 32:7 and a volume ratio of 31:4. The materials were gently mixed (but not ground) in the mortar, whereby the Pb foils remained as solid pieces throughout. The Ce-Pb mixture was pelletized at 1.5 metric tons (nominal pressure of 0.6 GPa) in a dedicated pellet press yielding a final pellet weight of ~385 mg. Next, the pellet was transferred into an encapsulated gasket. No pressure medium was added to avoid any form of contamination, especially with water and other potential hydrogen sources. Still in the glove box, the gasket was sealed at 5 metric tons. Within ~2 min, the pre-deformed gasket was transferred inside a sealed He-filled bag from the glove box to the PE press, where a load of 5 metric tons was applied within ~1 min.

Time-of-flight neutron diffraction was conducted on the SNAP beamline at the Spallation Neutron Source (Oak Ridge, Tennessee). The beamline was operated using the high-flux focusing-mirror guide, with the choppers set at a center wavelength of *λ*  = 2.1 Å. The two detector banks were placed at different 2*θ* positions, 65^∘^ and 105^∘^, providing a balance of Q-range and resolution. Data were collected for typically 1 h per measurement, with some shorter runs of 10 min used only to extract lattice parameters. The data were reduced using SNAPRed reduction routines^71^ within Mantid Workbench^72^. Full Rietveld refinements (for all included phases) were performed using Topas v6^73^. Refinement of the crystal structures was performed simultaneously against the two detector-bank histograms using a single, common structure. The pressure was extracted from the Pb pressure marker using a parameterization of literature data for a pressure- and temperature-dependent equation of state of the face-centered cubic phase of Pb^74^. The strong texture, observed only in the orthorhombic phase during the room temperature compression, was accounted for using preferred orientation modeling in Topas v6. To avoid issues with correlation in the refinements, the Ce coordinate in this phase was fixed.

A total of three loadings were performed. The first run was compression to 7.9 GPa at room temperature. The second and third runs included low-temperature conditions as well as temperature- and pressure-cycling. Specifically, the second run focused on compression at 100 K to a maximum load of 83 metric tons. This was followed by a temperature cycle that included heating to 285 K, cooling back to 85 K, followed by repeat heating to 295 K. The third run confirmed the compression pathway seen in the second run through compression at 120 K to a maximum load of 87 metric tons. There, the cell was heated to 285 K, and a pressure-cycle followed, i.e., the cell was decompressed to 60 metric tons and then re-compressed to 87 metric tons. The full details of these pressure–temperature cycles and pathways are shown in the SI Fig. S3.

## Supplementary information

Supplementary Information

Description of Additional Supplementary Information

Supplementary Data 1

Supplementary Data 2

Transparent Peer Review File

## Source data

Source Data

## Data Availability

The raw neutron data relating to this study are available online^75^. Crystallographic data for the *α*^″^ coexisting with *γ* at 120 K and 6.44(14) GPa (pellet 3) have been deposited at the Cambridge Crystallographic Data Centre, under deposition numbers CCDC 2526171 and 2526172. Copies of the data can be obtained free of charge via https://www.ccdc.cam.ac.uk/structures/, and are also attached to this manuscript as Supplementary Data 2. The reported goodness of fit for this powder-CIF is typical of that expected for data collected on the instrument in the Paris–Edinburgh press. Source data are provided with this paper.
