# Supplementary material for: The impact of metastability on the high-pressure behavior of cerium
Source: Nat Commun. 2026 Jul 13;17:6133. doi: 10.1038/s41467-026-74329-w (PMC13365817; doi:10.1038/s41467-026-74329-w)

Supplementary Information: The impact of metastability on the high-pressure behavior of cerium

Christopher J. Ridley,[1,*] Alice I. Smith,[2] Luke L. Daemen,[1,3] and Bianca Haberl[1,4]

[1]*Neutron Scattering Division, Neutron Sciences Directorate, Oak Ridge National Laboratory, Oak Ridge, TN 37830, USA*

[2]*Materials Science and Technology Division, Los Alamos National Laboratory, Los Alamos, NM 87545, USA*

[3]*University of California, Riverside, Riverside, CA 92521, USA*

[4]*Department of Materials Physics, Research School of Physics, The Australian National University, Canberra, ACT 2601, Australia*

(Dated: June 3, 2026)

* Corresponding author: ridleycj@ornl.gov

SUPPLEMENTARY NOTE 1: BULK MODULUS

Comparing the bulk modulus of α-Ce; our reported B_0 = 11.1(2) GPa, B' = 10.43(13) are very different to those from Olsen *et al.* [1] B_0 = 20(3) GPa, B' = 5.5(5) and Jeong *et al.* [2] $B_0 \approx 25$ GPa. However, many of these studies use different functional forms of the equation of state, or simply use finite differences between single data-points. To clarify this comparison, we have re-fitted these literature data using 2nd and 3rd order Birch-Murnaghan and Rydberg-Vinet equations of state (see Table S1). With the 2nd order, our bulk modulus is in broad agreement with Olsen *et al.* [1] and Jeong *et al.* [2] whereas with the 3rd order, our refined values are in better agreement with the original work of Zachariasen and Ellinger [3], who observe a similarly large curvature in the volume-pressure curve.

TABLE S1. Parameters obtained for Rydberg-Vinet (RM) and Birch-Murnaghan (BM) equation of states fits. For both cases, fits were performed with B_0' fixed to 4 and included into the fit. The same procedure was applied to literature data taken from Zachariasen and Ellinger [3], Olsen *et al.* [1], Jeong *et al.* [2] and Ma *et al.* [4].

	RV, $B_0' = 4$		BM, $B_0' = 4$		RV			BM		
	B_0 [GPa]	V_0 Å^3	B_0 [GPa]	V_0 Å^3	B_0 [GPa]	V_0 Å^3	B_0'	B_0 [GPa]	V_0 Å^3	B_0'
this work	24.3(8)	114.2(4)	23.17(4)	114.31(2)	11.1(2)	117.46(9)	10.43(13)	8.6(4)	118.3(2)	15.5(7)
Zachariasen and Ellinger [3]	23(1)	113.8(7)	23(2)	113.8(8)	6(1)	118.7(9)	14.6(9)	8(4)	117(2)	17(9)
Olsen *et al.* [1]	22(1)	116.1(8)	21(2)	116.2(8)	7(8)	122(7)	13(6)	-	-	-
Jeong *et al.* [2]	20.8(6)	115.7(3)	20.7(6)	115.7(2)	26(3)	114.9(5)	1(2)	25(3)	114.9(5)	1.8(1.3)
Ma *et al.* [4]	26.6(9)	114.0(3)	26.3(9)	114.1(3)	29(6)	113.7(9)	3(2)	29(5)	113.7(9)	3(2)

SUPPLEMENTARY NOTE 2: REMNANT γ-Ce OR IMPURITY?

Despite extensive purification of the Ce used in this study, we have considered the possibility that the residual γ-Ce observed at high pressure originates from an impurity phase with modified lattice parameters; this possibility is discussed here. Neutrons are highly sensitive to oxide and hydride content. Pb foil is included as the pressure marker, so PbO ($P4/nmm$) could plausibly be present in the patterns. Trial inclusion of this phase, with adjusted lattice parameters, was unable to account for the observed peaks. Both CeO_2 ($a \approx 5.427$ Å) and CeO ($a \approx 5.089$ Å) have the same symmetry as α/γ-Ce ($Fm\bar{3}m$) [5]. We use the known bulk modulii of these two oxides to estimate where the principal reflections should be observed [6, 7] if present. It is clear that CeO_2 is absent, with an initially larger lattice parameter and high bulk modulus. While it seems implausible that CeO could be present, given that it requires high pressure/temperature synthesis conditions [5, 8], it has a very similar lattice parameter to Ce at these pressures. The Ce-(200) reflection is observed at 2.5407(5) Å at 0.85 GPa, greater than expected from the equation of state for CeO [7]. At higher pressures, the observed and expected unit-cell volume diverges further (see Figure S1). Dmitriev *et al.* [9] state that they observe CeO under pressure, without heating, suggesting that it may have been present in the sample before loading. Leger *et al.* [5] only observe the formation of CeO under combined high pressure/temperature conditions, when mixed with CeO_2 (which as noted above, is clearly absent from our measurements). Additionally, the lattice parameters reported by Dmitriev *et al.* [9] are in much better agreement with out own observations, than with those reported for CeO from Leger [7] (see Figure S1). We therefore suggest that we clearly observe remnant γ-Ce, with an enhanced bulk modulus, and not an oxide, and this may have been observed but not realized in previous work [9]. A similar process allows us to quickly eliminate CeH_2 as a potential impurity from the present study.

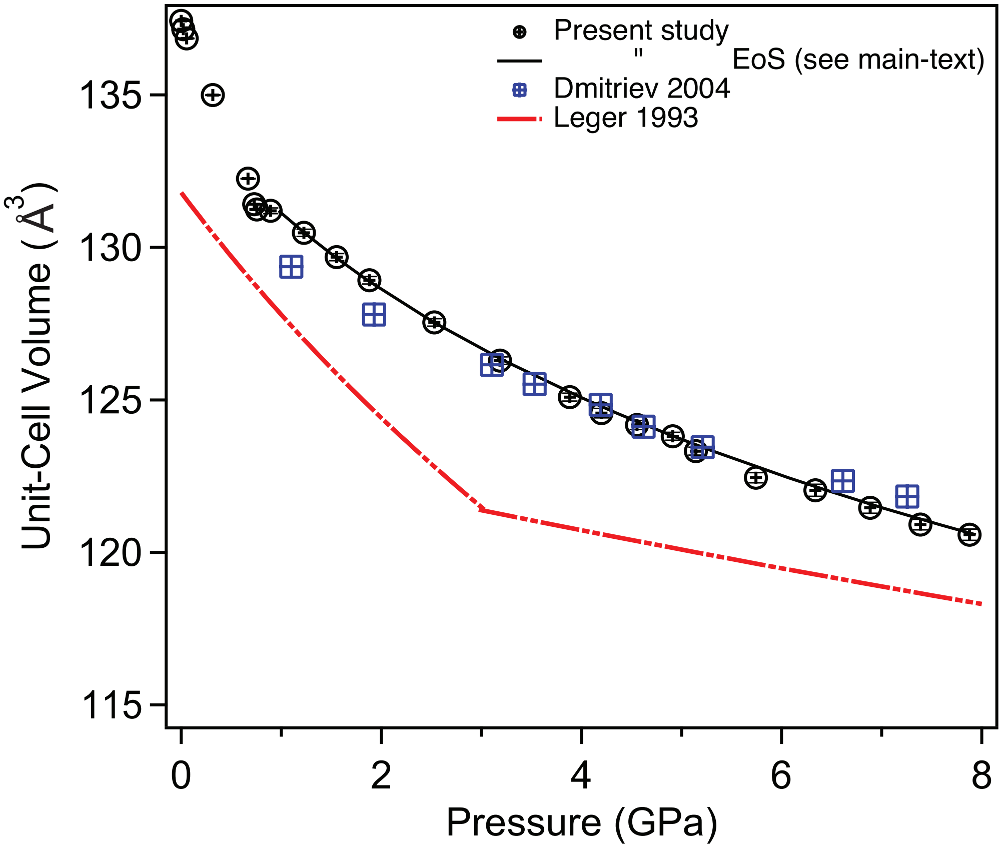

FIG. S1. Comparing the unit-cell volume evolution of the remnant γ-Ce with that expected for CeO. The black circles are from the present study, the black line is the fit to the equation-of-state, discussed in the main text. The blue square markers are extracted from Dmitriev *et al.* [9] and the red line is calculated from Leger [7]. The central values and errors were extracted from the peak fitting. The error bars are obscured by the symbols in some instances. Source data are provided as a Source Data file.

SUPPLEMENTARY NOTE 3: EXPERIMENTAL DETAILS FROM OUR OWN WORK AND LITERATURE

Three different pellets were prepared for the three runs. Following gentle mixing of Ce and Pb in a mortar and pelletization applying a nominal pressure of 0.6 GPa, the pellets were retrieved and placed back in the mortar for inspection. Photos of the three resulting pellets inside the mortar are shown in Figure S2. The pellets were then sealed into the encapsulated TiZr gaskets, with an example of such a sealed sample/gasket assembly shown in Figure S2(c). Note that the entire preparation was performed within a glovebox with the photos taken from outside.

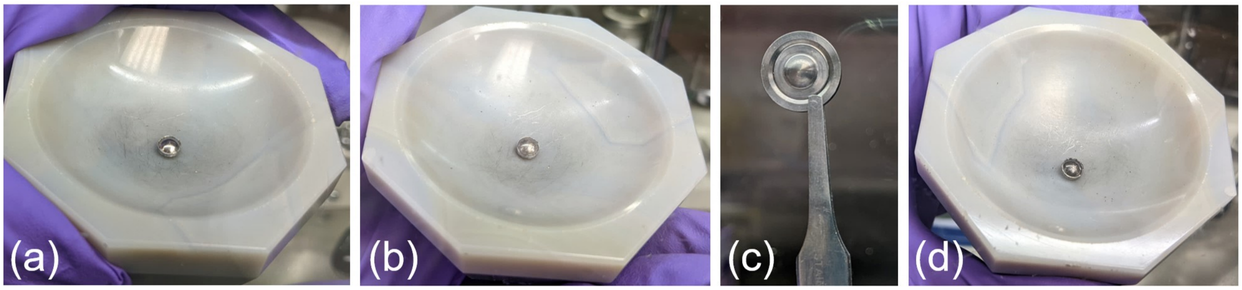

FIG. S2. Snapshots taken during pellet preparation inside the glove box. (a) Pellet used in run #1, prepared from 320 mg Ce and 70 mg Pb, (b) pellet used in run #2, prepared from 320 mg Ce and 72 mg Pb, (c) same pellet after sealing inside the gasket, and (d) pellet used in run #2, prepared from 321.5 mg Ce and 72.5 mg Pb.

These three pellets were used for room temperature compression in run #1 and two low-temperature compression cycles in runs #2 and #3. Temperature- and pressure-cycling was added in the last two runs to better elucidate phase behaviors. The detailed load and temperature conditions applied to the samples are shown in Figure S3.

A compilation of key experimental parameters used in our own work and key literature report with a focus on the α' vs. α'' formation is given in Table S2. The dependence of α'' vs. α' on the sample preparation method has been observed and well described by McMahon and Nelmes [10].

Further, no dependence on the hydrostatic conditions used in these past studies can be discerned from the table. Specifically, while McMahon and Nelmes [10] can control the phase transition sequence through the sample preparation method in the absence of a pressure transmitting medium (PTM), they also performed test experiments with 4:1

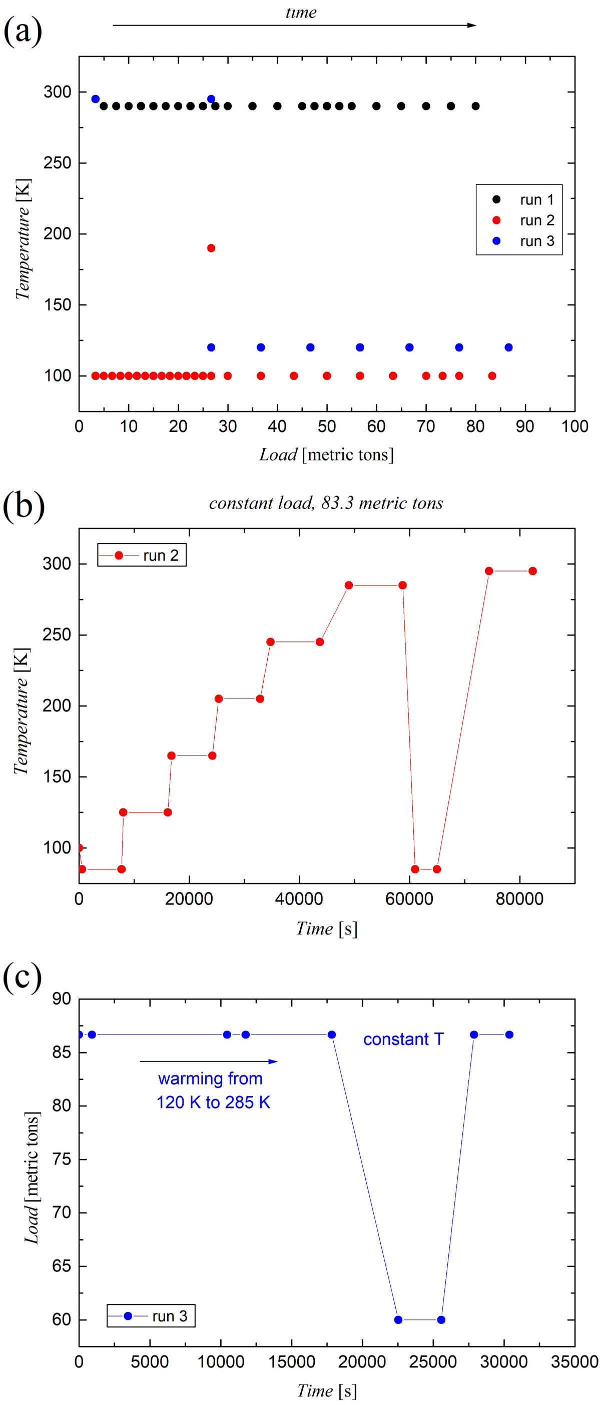

FIG. S3. (a) Schematic of the three experimental runs. Run 1 was a room-temperature compression to a maximum load of 80 metric tons. Run 2 cooled to 100 K followed by compression. At a load of 27 metric tons, the cell was heated to 190 K and cooled back to 100 K for compression to 83 metric tons. At this load and pressure, the sample was then temperature cycled, as shown in (b). First it was heated to 285 K, then cooled back to 85 K, then heated to 295 K. Run 3 was compressed to 27 metric tons at room temperature, then cooled to 120 K for further compression to 87 metric tons. (c) At this load, the cell was heated to 285 K and then decompressed to 60 metric tons, and re-compressed to 87 metric tons. Source data are provided as a Source Data file.

methanol:ethanol, which did not alter phase transition sequences [10]. This is consistent with the later study by Dmitriev *et al.* [9] where both, pressurization without PTM and with silicone oil yielded the monoclinic α'' phase.

There is however, a key difference between these two studies above and our own study: The use of diamond anvil cells with flat anvils vs. toroidal anvils with cupped recessions for samples space as used in the Paris-Edinburgh press here. In the absence of a PTM, compression with flat anvils yields strong deviatoric stresses. Measurements on a diamond cell on NaCl pressurized to 1 GPa show a gradient of 0.2 GPa at these pressures already [11]. These stresses are tempered through a cupped sample space as used with the toroidal anvils in a Paris-Edinburgh press. Indeed, measurements on NaCl compressed to 6 GPa using such cupped anvils suggest that pressure gradients in the equatorial gap between the anvils could be as low as 1% [12]. The bulk and shear moduli of NaCl are very similar to those of Ce in the γ phase and similar distributions are thus likely. Further, in the present experiment, opaque cBN anvils and B_4C slits defining the incident beam were used together with a placement of the cell in through-gasket geometries for incident beam and scattered beam. Thus, only this equatorial part of the sample is illuminated and accordingly we expect to observe very low pressure gradients only.

Thus, the samples pressurized here most likely experience significantly less deviatoric stresses than samples compressed without PTM in a diamond cell. Further, a number of experiments used silicone oil, which does see an increase in pressure inhomogeneity at pressures above ~2.5 GPa [13]. Experiments using this PTM in a DAC may thus also yield higher deviatoric stresses than observed here. In terms of hydrostaticity, it could well be that the present experiment is best comparable to past diamond cell experiments using 4:1 methanol:ethanol and of course the toroidal anvils used by Tsiok and Khvostantsev [14].

Beyond hydrostaticity a key experimental difference is the reported purity of the Ce samples used. Where provided, literature reports all state 99.9% or 99.99% purity. The samples used in the present study underwent specific purification to a level of 99.999% purity.

Overall, the experiment here aimed to co-optimize for sample purity (i.e. sample handling in the glove box, no PTM that may release water) and hydrostaticity (given the high pressure/low temperature extremes used here, PTM would freeze). In the absence of helium gas loading that would not compromise purity, the current use of cupped toroidal anvils

without PTM appears a suitable experimental choice.

Finally, while not listed in the table, it is noteworthy that sample volume/mass used here is quite different to diamond anvil cells experiments. The starting sample mass of 320 mg of Ce (plus some Pb) is several orders of magnitude larger than the sample mass used in the diamond cell, which is typically in the order of 0.01 mg. The behaviors seen here therefore reflect bulk phase behaviors more closely.

TABLE S2. Resulting high-pressure polymorph above ∼5 GPa and the experimental conditions described in literature during room temperature compression. Key parameters are the purity and form of the starting Ce material, the pressure transmitting medium (PTM) and any notes on the presence of hydrogen during experiment or in resulting data.

	high-pressure phase	purity	form	PTM	hydrogen presence
this work	both	99.999%	filings	none	sample stored in Ar
Zachariasen and Ellinger [3]	both	high purity	unknown	none?	
Olsen *et al.* [1]	α''	99.9%	fine powder	4:1 ME	CeH_2 noted by [10]
Gu *et al.* [15]	both	99.99%	chips	4:1 ME	CeH_2 noted by [10]
Zhao and Holzapfel [16]	both	99.9%	chips	mineral oil	sample stored in Ar
McMahon and Nelmes [10]	α''	99.9%	filings	none (and 4:1 ME)	CeH_2 observed
——	α''	99.99%	filings	none (and 4:1 ME)	CeH_2 observed
——	α'	99.9%	chips	none	
Tsiok and Khvostantsev [14]	both	99.99%	large piece	polysiloxane	none
Jeong *et al.* [2]	-	99.99%	solid rod	4:1 ME	
Ma *et al.* [4]	α''	99.9%	cuts	silicone oil	CeH_2 observed
Dmitriev *et al.* [9]	α''	99.9%	lumps	none and silicone oil	none, CeO instead

SUPPLEMENTARY NOTE 4: DETAILED RESULTS OF RIETVELD REFINEMENTS

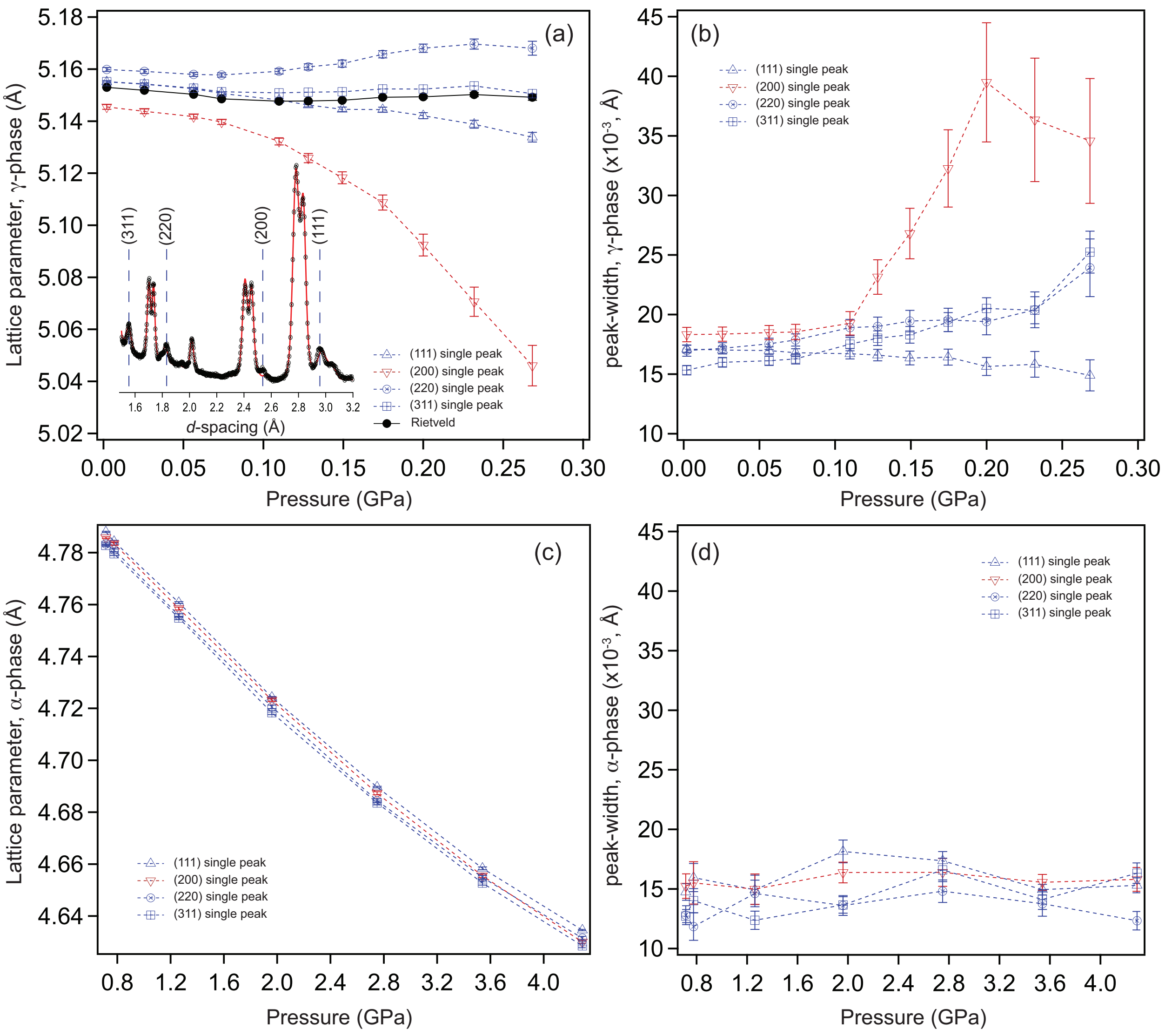

FIG. S4. Low pressure lattice behaviour (a) and peak-shape changes (b) of γ phase at 100 K. The Rietveld refined lattice parameter (taking all peaks from γ into account) appears to show an inflexion and small increase in volume above 0.1 GPa. The fit (inset) shows the four principal reflections (*hkl*s indicated) that constitute γ at 0.27 GPa, showing that observed vs fitted peak positions are off with some complex *hkl*-dependence. If each reflection is fitted as a single peak, unconstrained by symmetry or intensity, then an effective lattice parameter can be extracted for each *hkl*. When overlaid with the lattice parameter determined from Rietveld, it is clear that each *hkl* is influencing the average lattice-parameter very differently. Similarly, an anomalous broadening of the (200) reflection is observed. This process was repeated for the isostructural α phase from the same pellet at 100 K (c,d), which does not show *hkl*-dependent shifts or peak broadening over a wider pressure range. Source data are provided as a Source Data file. The central values and errors were extracted from the peak fitting. The error bars are obscured by the symbols in some instances.

TABLE S3. Rietveld refined lattice parameters for each of the observed phases of Ce discussed in the main manuscript, determined using neutron diffraction data collected from pellet 1 within the Paris-Edinburgh press.

**Pellet 1, 300 K**	γ (cubic)	α (cubic)	β (hex)		α' (ortho)			α'' (mono)			
Pressure(GPa)	a (Å)	a (Å)	a (Å)	c (Å)	a (Å)	b (Å)	c (Å)	a (Å)	b (Å)	c (Å)	β (deg)
0.000(6)	5.16051(13)		3.664(2)	11.872(13)							
0.010(6)	5.15709(14)		3.667(2)	11.94(1)							
0.054(6)	5.1533(2)		3.659(2)	11.851(15)							
0.316(6)	5.1299(2)		3.660(2)	11.817(12)							
0.667(6)	5.09489(11)		3.657(3)	11.68(2)							
0.730(6)	5.08411(13)	4.8204(2)									
0.756(6)	5.0819(3)	4.8147(1)									
0.893(7)	5.0814(12)	4.80114(8)									
1.226(8)	5.0720(2)	4.77534(8)									
1.553(9)	5.0617(2)	4.75347(8)									
1.882(12)	5.0518(2)	4.73473(8)									
2.53(2)	5.0337(2)	4.70251(8)									
3.18(3)	5.0171(2)	4.67440(9)									
3.88(4)	5.0012(2)	4.6477(1)									
4.20(5)	4.9942(2)	4.63645(11)									
4.55(5)	4.9890(2)	4.62497(11)									
4.91(6)	4.9841(2)	4.61372(13)									
5.14(7)	4.9775(2)	4.6069(2)			3.0855(4)	6.0161(9)	5.2328(9)				
5.74(8)	4.9657(2)	4.5891(8)			3.0721(3)	5.9959(7)	5.2186(7)				
6.34(9)	4.960(3)	4.572(6)			3.0605(3)	5.9748(7)	5.2044(7)	5.825(6)	3.166(2)	5.655(7)	113.10(8)
6.89(12)	4.9524(2)				3.0502(3)	5.9573(8)	5.1923(8)	5.811(7)	3.156(2)	5.631(8)	113.15(9)
7.39(13)	4.9449(2)				3.0411(3)	5.9419(9)	5.1812(9)	5.806(7)	3.146(3)	5.614(9)	113.2(1)
7.9(2)	4.9403(2)				3.0324(4)	5.9286(9)	5.1711(10)	5.795(8)	3.132(3)	5.60(1)	113.24(10)

TABLE S4. Rietveld refined lattice parameters for each of the observed phases of Ce discussed in the main manuscript, determined using neutron diffraction data collected from pellet 2 within the Paris-Edinburgh press.

**Pellet 2**		γ (cubic)	α (cubic)	β (hex)		α'' (mono)			
Temperature (K)	Pressure(GPa)	a (Å)	a (Å)	a (Å)	c (Å)	a (Å)	b (Å)	c (Å)	β (deg)
100	0.000(6)	5.1530(2)		3.654(4)	12.08(3)				
100	0.026(6)	5.1519(2)		3.653(4)	12.06(2)				
100	0.056(6)	5.1503(2)		3.651(4)	12.10(2)				
100	0.074(6)	5.1486(2)		3.653(3)	12.108(17)				
100	0.109(6)	5.1477(3)	4.8323(8)	3.657(2)	12.134(14)				
100	0.128(7)	5.1477(3)	4.8302(6)	3.660(2)	12.134(13)				
100	0.149(7)	5.1480(4)	4.8283(5)	3.657(2)	12.147(13)				
100	0.175(7)	5.1492(4)	4.8268(4)	3.658(2)	12.140(12)				
100	0.199(7)	5.1493(6)	4.8245(3)	3.659(2)	12.137(11)				
100	0.232(7)	5.1502(8)	4.8218(3)	3.659(2)	12.135(12)				
100	0.268(7)	5.1492(12)	4.8189(2)	3.656(2)	12.110(11)				
heated to 180									
100	0.714(8)	5.064(4)	4.78223(11)						
100	0.776(11)	5.061(4)	4.7792(2)						
100	1.262(11)	5.038(6)	4.7550(2)						
100	1.96(2)	5.014(6)	4.7188(2)						
100	2.75(2)	4.963(5)	4.6834(2)						
100	3.54(3)	4.965(6)	4.6529(2)						
100	4.29(5)	4.964(5)	4.6272(2)						
100	5.03(6)	4.965(3)	4.6043(3)						
100	5.12(6)	4.972(2)	4.6003(3)			5.858(6)	3.216(3)	5.662(4)	113.37(7)
100	5.44(7)	4.967(2)	4.5909(3)			5.843(3)	3.1939(13)	5.647(2)	113.18(4)
100	5.73(8)		4.5808(7)			5.832(3)	3.1788(9)	5.633(2)	113.06(3)
100	6.32(9)					5.821(3)	3.1590(9)	5.606(2)	112.99(3)
85	6.31(9)					5.821(2)	3.1586(8)	5.604(2)	112.97(3)
125	6.80(11)					5.816(3)	3.1490(9)	5.591(2)	113.03(3)
165	7.28(13)					5.815(3)	3.1393(10)	5.583(3)	113.10(3)
205	7.9(2)					5.814(4)	3.1293(12)	5.580(3)	113.21(4)
245	8.2(2)					5.815(5)	3.1244(14)	5.583(4)	113.19(5)
285	8.4(2)					5.806(7)	3.126(3)	5.602(7)	112.91(8)

TABLE S5. Rietveld refined lattice parameters for each of the observed phases of Ce discussed in the main manuscript, determined using neutron diffraction data collected from pellet 3 within the Paris-Edinburgh press. Note that while γ is present, it is only as a trace amount, and the lattice parameters could only be extracted for the longer data collections.

**Pellet 3**		γ (cubic)	α (cubic)	α'' (mono)			
Temperature (K)	Pressure(GPa)	a (Å)	a (Å)	a (Å)	b (Å)	c (Å)	β (deg)
295	0.002(6)	5.1650(2)					
295	1.060(6)	*trace*	4.78485(11)				
295	1.126(5)	*trace*	4.77982(1)				
120	0.769(6)	*trace*	4.77306(7)				
120	1.359(8)	*trace*	4.74255(9)				
120	2.46(2)	*trace*	4.68816(9)				
120	3.47(3)	*trace*	4.64879(11)				
120	4.61(5)	*trace*	4.61113(11)				
120	5.55(7)	*trace*	4.5861(2)	5.841(3)	3.1737(6)	5.622(2)	113.03(2)
120	6.44(14)	4.937(5)		5.833(2)	3.1500(7)	5.5888(12)	113.08(2)

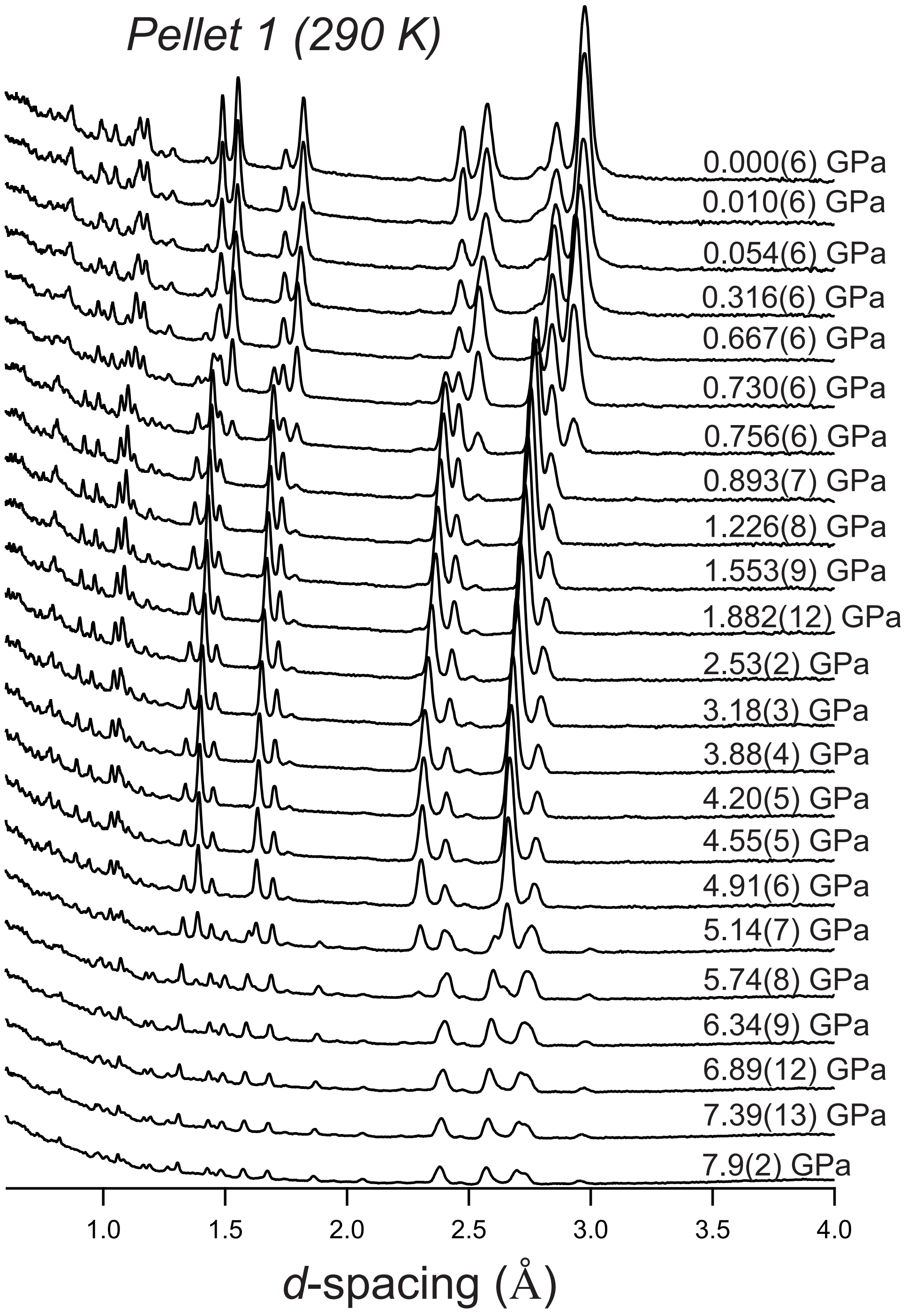

FIG. S5. Stack plot of neutron powder diffraction patterns collected from pellet 1, at room temperature, from within the Paris-Edinburgh Press. Source data are provided as a Source Data file.

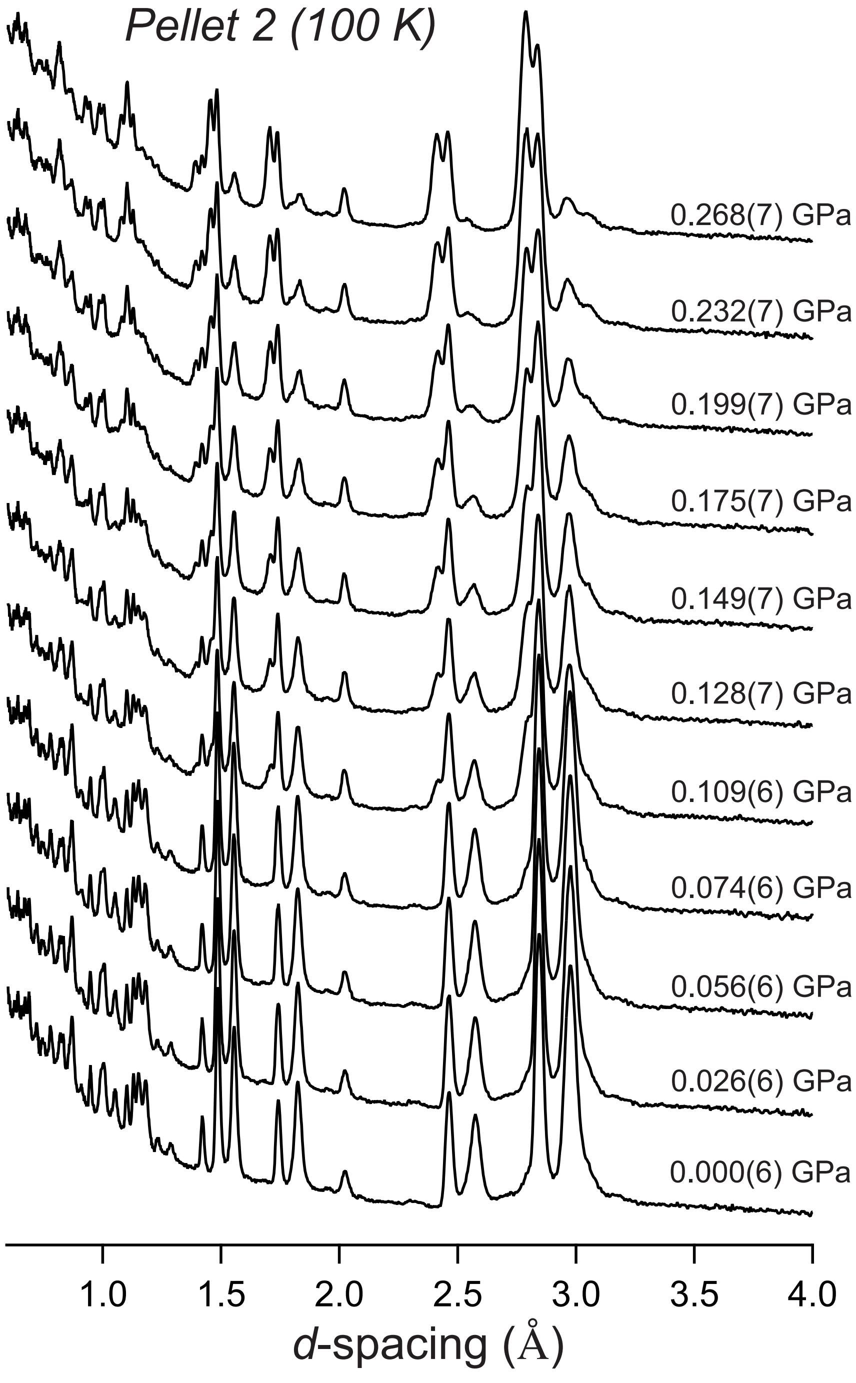

FIG. S6. Stack plot of neutron powder diffraction patterns collected from pellet 2, at room temperature, from within the Paris-Edinburgh Press, prior to heat cycling to 180 K. Source data are provided as a Source Data file.

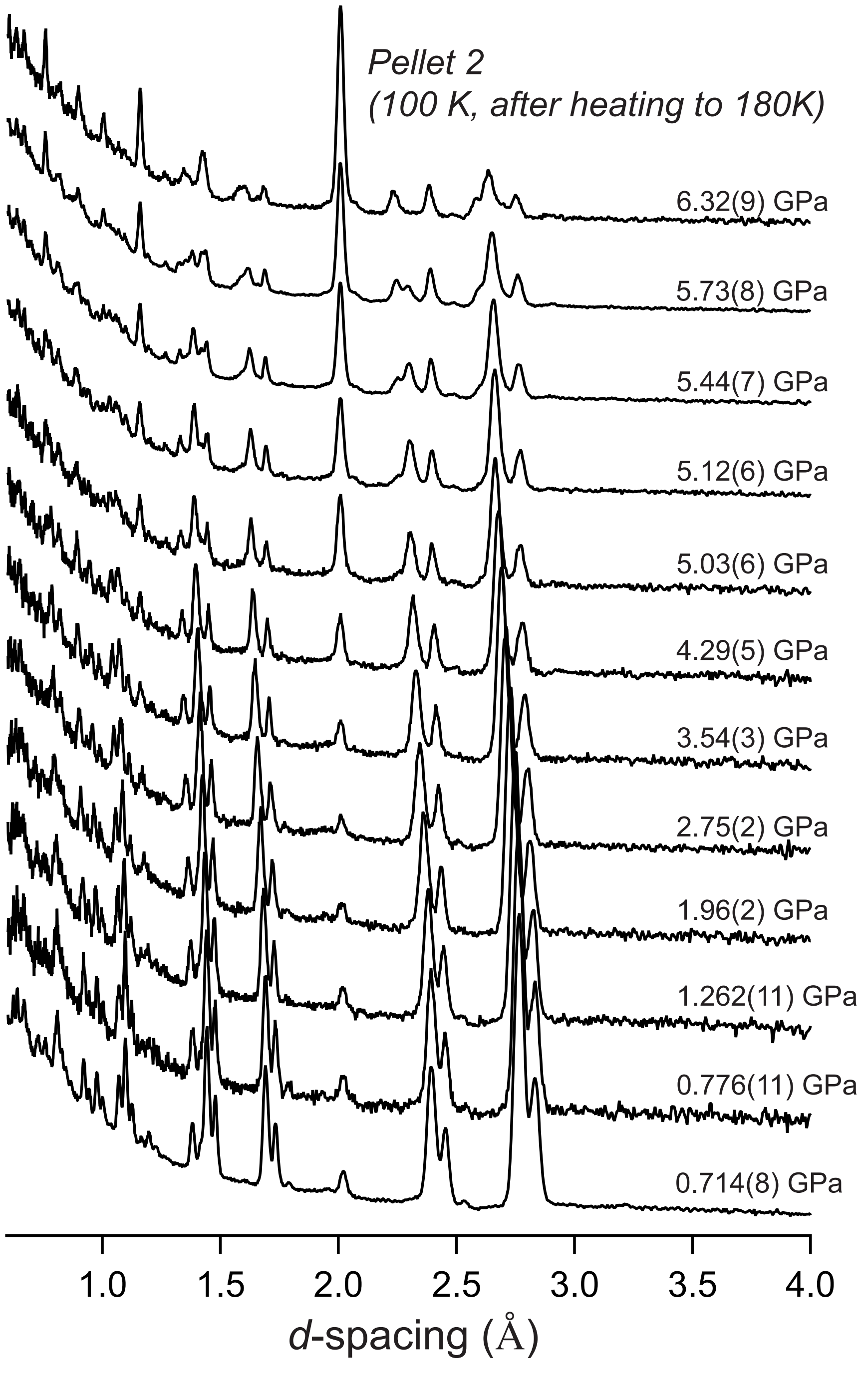

FIG. S7. Stack plot of neutron powder diffraction patterns collected from pellet 2, at room temperature, from within the Paris-Edinburgh Press, post heat cycling to 180 K. Source data are provided as a Source Data file.

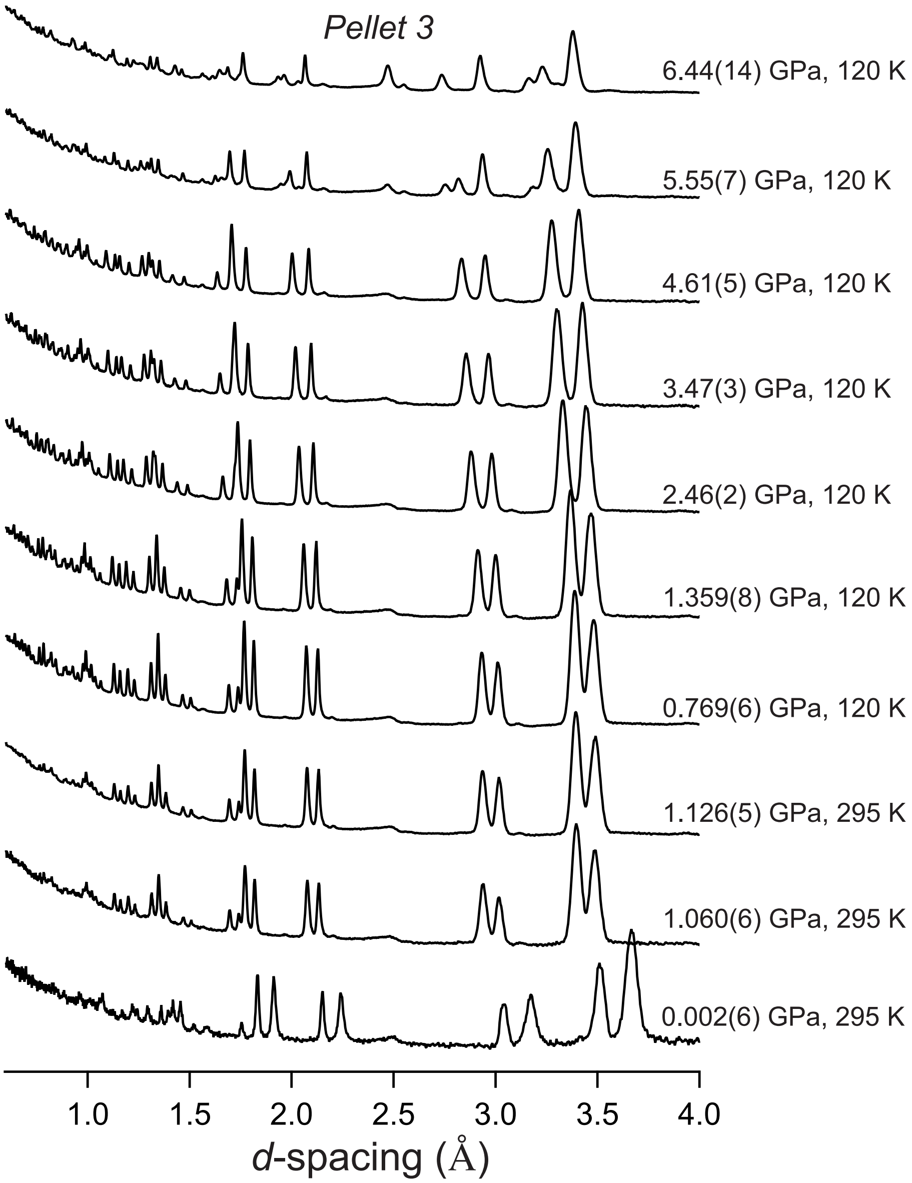

FIG. S8. Stack plot of neutron powder diffraction patterns collected from pellet 3, temperatures indicated, from within the Paris-Edinburgh Press. Source data are provided as a Source Data file.

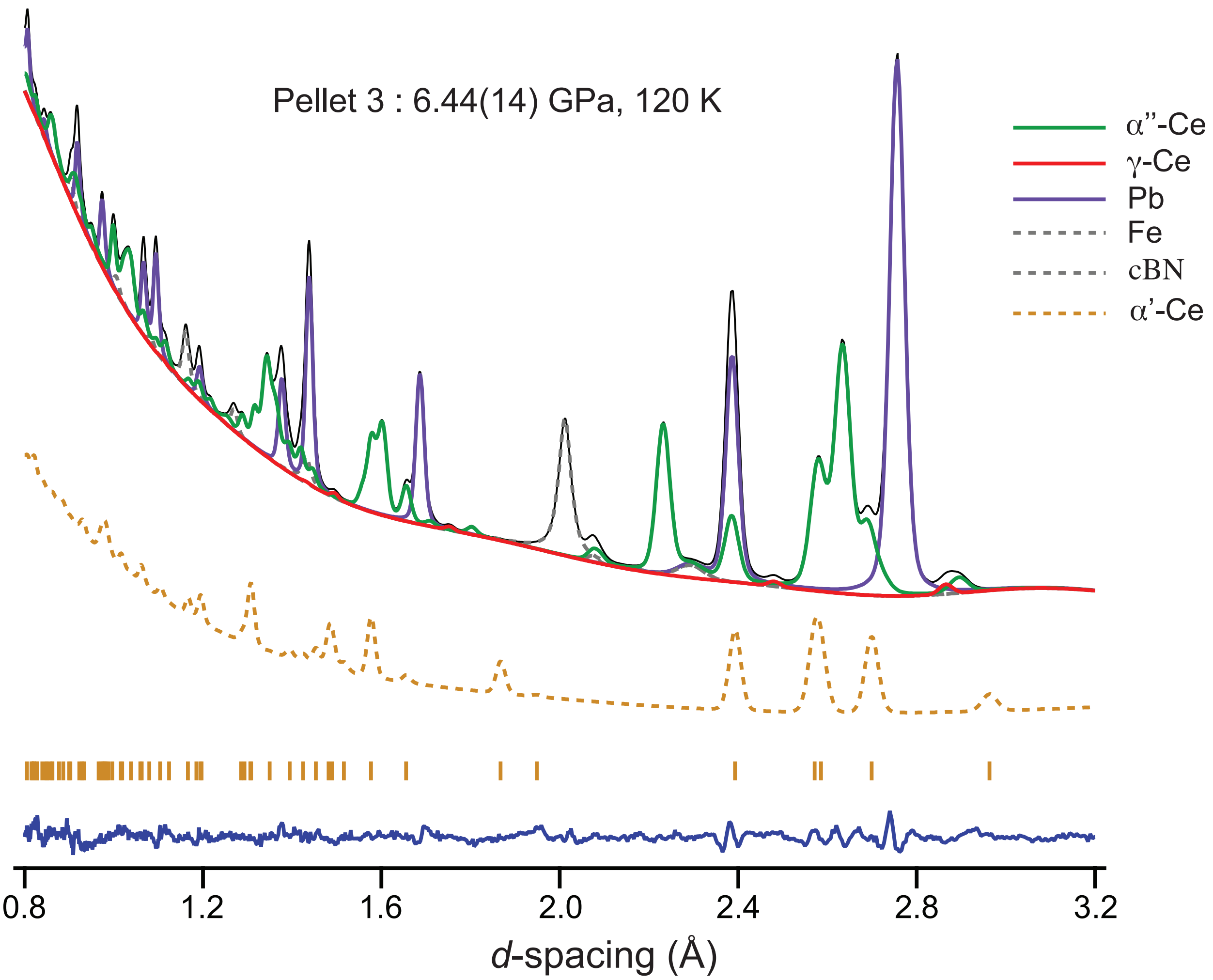

FIG. S9. Recreation of Figure 2(b) from the main manuscript, showing the individual contributions of each phase to the overall Rietveld calculation at 6.44(14) GPa and 120 K. Additionally, the simulated contribution from the absent α'-Ce is shown, along with the tick marks for this phase. This is simulated from Figure 2(a) in the main manuscript, 7.9(2) GPa and 295 K. The observed data points and overall calculated intensity are not shown, for clarity. The blue line is the residual to the overall fit. Source data are provided as a Source Data file.

SUPPLEMENTARY REFERENCES

[1] J. Olsen, L. Gerward, U. Benedict, and J.-P. Itié, Physica B+C **133**, 129 (1985).

[2] I. K. Jeong, T. W. Darling, M. J. Graf, T. Proffen, R. H. Heffner, Y. Lee, T. Vogt, and J. D. Jorgensen, Physical Review Letters **92**, 105702 (2004).

[3] W. H. Zachariasen and F. H. Ellinger, Acta Crystallographica Section A **33**, 155 (1977).

[4] C. Ma, X. Tan, Z.-y. Dou, J.-r. Jiang, B. Bai, H.-y. Zhu, P.-c. Zhang, and Q.-l. Cui, Journal of Alloys and Compounds **712**, 588 (2017).

[5] J. M. Leger, N. Yacoubi, and J. Loriers, Journal of Solid State Chemistry **36**, 261 (1981).

[6] L. Gerward, J. S. Olsen, L. Petit, G. Vaitheeswaran, V. Kanchana, and A. Svane, Journal of Alloys and Compounds **400**, 56 (2005).

[7] J. M. Leger, Physica B: Condensed Matter **190**, 84 (1993).

[8] J. M. Leger, N. Yacoubi, and J. Loriers, Materials Research Bulletin **14**, 1431 (1979).

[9] V. P. Dmitriev, A. Y. Kuznetsov, O. Bandilet, P. Bouvier, L. Dubrovinsky, D. Machon, and H.-P. Weber, Physical Review B **70**, 014104 (2004).

[10] M. I. McMahon and R. J. Nelmes, Physical Review Letters **78**, 3884 (1997).

[11] S. Usha Devi and A. Singh, Physica B+C **139-140**, 922 (1986).

[12] S. Klotz, J. M. Besson, and G. Hamel, High Pressure Research **26**, 277 (2006).

[13] S. Klotz, J.-C. Chervin, P. Munsch, and G. Le Marchand, Journal of Physics D: Applied Physics **42**, 075413 (2009).

[14] O. B. Tsiok and L. G. Khvostantsev, J. Exp. Theor. Phys. **93**, 1245 (2001).

[15] G. Gu, Y. K. Vohra, and K. E. Brister, Physical Review B **52**, 9107 (1995).

[16] Y. Zhao and W. B. Holzapfel, Journal of Alloys and Compounds **246**, 216 (1997).

checkCIF/PLATON report

You have not supplied any structure factors. As a result the full set of tests cannot be run.

THIS REPORT IS FOR GUIDANCE ONLY. IF USED AS PART OF A REVIEW PROCEDURE FOR PUBLICATION, IT SHOULD NOT REPLACE THE EXPERTISE OF AN EXPERIENCED CRYSTALLOGRAPHIC REFEREE.

No syntax errors found. CIF dictionary Interpreting this report

Datablock: Pb

```
Bond precision:    = 0.0000 A              Wavelength=0

Cell:             a=4.75712(14)     b=4.75712(14)     c=4.75712(14)
                  alpha=90          beta=90           gamma=90
Temperature:      120 K

                 Calculated                 Reported
Volume           107.655(10)                107.655(10)
Space group      F m -3 m                   Fm-3m
Hall group       -F 4 2 3                   -F 4 2 3
Moiety formula   Pb                         Pb
Sum formula      Pb                         Pb
Mr               207.20                     207.20
Dx,g cm-3        12.784                     12.784
Z                4                          4
Mu (mm-1)        0.000                      0.000
F000             37.6                       0.0
F000'            327.80
h,k,lmax
Nref
Tmin,Tmax
Tmin'

Correction method= Not given
```

Data completeness= Theta(max)=

R(reflections)= wR2(reflections)=
S = Npar=

The following ALERTS were generated. Each ALERT has the format
test-name_ALERT_alert-type_alert-level.
Click on the hyperlinks for more details of the test.

Alert level A

```
PLAT721_ALERT_1_A Bond    Calc     0.00000, Rep 3.36380(10) Dev...       3.36 Ang.
             PB1      -PB1           1_555   1_555 ........     #    1 Check
```

Author Response: The reported atom separation is correct.

Alert level G

```
PLAT304_ALERT_4_G Non-Integer Number of Atoms in ..... (Resd    1)       0.02 Check
PLAT898_ALERT_4_G Second Reported H-M Symbol in CIF Ignored ......          ! Check
```

---

1 **ALERT level A** = Most likely a serious problem - resolve or explain
0 **ALERT level B** = A potentially serious problem, consider carefully
0 **ALERT level C** = Check. Ensure it is not caused by an omission or oversight
2 **ALERT level G** = General information/check it is not something unexpected

1 ALERT type 1 CIF construction/syntax error, inconsistent or missing data
0 ALERT type 2 Indicator that the structure model may be wrong or deficient
0 ALERT type 3 Indicator that the structure quality may be low
2 ALERT type 4 Improvement, methodology, query or suggestion
0 ALERT type 5 Informative message, check

Datablock: Cerium_alphaPP

Bond precision: Ce-Ce = 0.0086 A Wavelength=0

Cell:	a=5.8351(14)	b=3.1481(4)	c=5.5862(8)
	alpha=90	beta=113.032(12)	gamma=90
Temperature:	120 K		

	Calculated	Reported
Volume	94.44(3)	94.43(3)
Space group	C 2/m	C2/m
Hall group	-C 2y	-C 2y
Moiety formula	Ce	Ce
Sum formula	Ce	Ce
Mr	140.12	140.12
Dx,g cm-3	9.855	9.855
Z	4	4
Mu (mm-1)	0.000	0.000
F000	19.4	0.0
F000'	231.92	
h,k,lmax		
Nref		
Tmin,Tmax		
Tmin'		

Correction method= Not given

Data completeness= Theta(max)=

R(reflections)= wR2(reflections)=
S = Npar=

The following ALERTS were generated. Each ALERT has the format
test-name_ALERT_alert-type_alert-level.
Click on the hyperlinks for more details of the test.

Alert level G

PLAT004_ALERT_5_G Polymeric Structure Found with Maximum Dimension 3 Info

---

0 **ALERT level A** = Most likely a serious problem - resolve or explain
0 **ALERT level B** = A potentially serious problem, consider carefully
0 **ALERT level C** = Check. Ensure it is not caused by an omission or oversight
1 **ALERT level G** = General information/check it is not something unexpected

0 ALERT type 1 CIF construction/syntax error, inconsistent or missing data
0 ALERT type 2 Indicator that the structure model may be wrong or deficient
0 ALERT type 3 Indicator that the structure quality may be low
0 ALERT type 4 Improvement, methodology, query or suggestion
1 ALERT type 5 Informative message, check

Datablock: Steel

Bond precision:	Fe-Fe = 0.0011 A	Wavelength=0	
Cell:	a=2.8592(11)	b=2.8592(11)	c=2.8592(11)
	alpha=90	beta=90	gamma=90
Temperature:	120 K		

	Calculated	Reported
Volume	23.37(3)	23.37(3)
Space group	I m -3 m	Im-3m
Hall group	-I 4 2 3	-I 4 2 3
Moiety formula	Fe	Fe
Sum formula	Fe	Fe
Mr	55.85	55.85
Dx,g cm-3	7.937	7.935
Z	2	2
Mu (mm-1)	0.000	0.000
F000	18.9	0.0
F000'	51.98	
h,k,lmax		
Nref		
Tmin,Tmax		
Tmin'		

Correction method= Not given

Data completeness= Theta(max)=

R(reflections)= wR2(reflections)=

S = Npar=

The following ALERTS were generated. Each ALERT has the format
test-name_ALERT_alert-type_alert-level.
Click on the hyperlinks for more details of the test.

Alert level G

PLAT004_ALERT_5_G Polymeric Structure Found with Maximum Dimension 3 Info
PLAT898_ALERT_4_G Second Reported H-M Symbol in CIF Ignored ...... ! Check

---

0 **ALERT level A** = Most likely a serious problem - resolve or explain
0 **ALERT level B** = A potentially serious problem, consider carefully
0 **ALERT level C** = Check. Ensure it is not caused by an omission or oversight
2 **ALERT level G** = General information/check it is not something unexpected

0 ALERT type 1 CIF construction/syntax error, inconsistent or missing data

0 ALERT type 2 Indicator that the structure model may be wrong or deficient
0 ALERT type 3 Indicator that the structure quality may be low
1 ALERT type 4 Improvement, methodology, query or suggestion
1 ALERT type 5 Informative message, check

Datablock: cBN

Bond precision: N- B = 0.0003 A Wavelength=0

Cell:	a=3.5971(7)	b=3.5971(7)	c=3.5971(7)
	alpha=90	beta=90	gamma=90
Temperature:	120 K		

	Calculated	Reported
Volume	46.54(3)	46.54(3)
Space group	F -4 3 m	F-43m
Hall group	F -4 2 3	F -4 2 3
Moiety formula	B N	B N
Sum formula	B N	B N
Mr	24.82	24.82
Dx,g cm-3	3.542	3.542
Z	4	4
Mu (mm-1)	0.000	0.000
F000	58.6	0.0
F000'	47.97	
h,k,lmax		
Nref		
Tmin,Tmax		
Tmin'		

Correction method= Not given

Data completeness= Theta(max)=

R(reflections)= wR2(reflections)=
S = Npar=

The following ALERTS were generated. Each ALERT has the format
test-name_ALERT_alert-type_alert-level.
Click on the hyperlinks for more details of the test.

Alert level G
PLAT004_ALERT_5_G Polymeric Structure Found with Maximum Dimension 3 Info

```
PLAT432_ALERT_2_G Short Inter X...Y Contact  B1       ..B1       .       2.54 Ang.
                                             1/2-x,-y,1/2+z  =     72_555 Check
PLAT432_ALERT_2_G Short Inter X...Y Contact  B1       ..B1       .       2.54 Ang.
                                             -y,1/2+x,1/2-z  =     80_555 Check
PLAT432_ALERT_2_G Short Inter X...Y Contact  B1       ..B1       .       2.54 Ang.
                                              x,1/2+y,1/2+z  =     73_555 Check
PLAT432_ALERT_2_G Short Inter X...Y Contact  B1       ..B1       .       2.54 Ang.
                                             -x,1/2-y,1/2+z  =     96_555 Check
PLAT432_ALERT_2_G Short Inter X...Y Contact  B1       ..B1       .       2.54 Ang.
                                              1/2+x,1/2+y,z  =     25_555 Check
PLAT432_ALERT_2_G Short Inter X...Y Contact  B1       ..B1       .       2.54 Ang.
                                              1/2+x,y,1/2+z  =     49_555 Check
PLAT432_ALERT_2_G Short Inter X...Y Contact  B1       ..B1       .       2.54 Ang.
                                             1/2+y,1/2-x,-z  =     41_555 Check
PLAT432_ALERT_2_G Short Inter X...Y Contact  B1       ..B1       .       2.54 Ang.
                                             1/2+y,-x,1/2-z  =     65_555 Check
PLAT432_ALERT_2_G Short Inter X...Y Contact  B1       ..B1       .       2.54 Ang.
                                             1/2-y,1/2+x,-z  =     32_555 Check
PLAT898_ALERT_4_G Second Reported H-M Symbol in CIF Ignored ......         ! Check
```

---

0 **ALERT level A** = Most likely a serious problem - resolve or explain
0 **ALERT level B** = A potentially serious problem, consider carefully
0 **ALERT level C** = Check. Ensure it is not caused by an omission or oversight
11 **ALERT level G** = General information/check it is not something unexpected

0 ALERT type 1 CIF construction/syntax error, inconsistent or missing data
9 ALERT type 2 Indicator that the structure model may be wrong or deficient
0 ALERT type 3 Indicator that the structure quality may be low
1 ALERT type 4 Improvement, methodology, query or suggestion
1 ALERT type 5 Informative message, check

Datablock: Cerium_gamma

Bond precision:	Ce-Ce = 0.0040 A	Wavelength=0	
Cell:	a=4.937(5)	b=4.937(5)	c=4.937(5)
	alpha=90	beta=90	gamma=90
Temperature:	120 K		

	Calculated	Reported
Volume	120.3(4)	120.3(4)
Space group	F m -3 m	F m -3 m
Hall group	-F 4 2 3	-F 4 2 3
Moiety formula	Ce	Ce
Sum formula	Ce	Ce
Mr	140.12	140.12
Dx,g cm-3	7.740	7.734
Z	4	4
Mu (mm-1)	0.000	0.000
F000	19.4	0.0
F000'	231.92	
h,k,lmax		
Nref		
Tmin,Tmax		
Tmin'		

Correction method= Not given

Data completeness= Theta(max)=

R(reflections)= wR2(reflections)=

S = Npar=

The following ALERTS were generated. Each ALERT has the format
test-name_ALERT_alert-type_alert-level.
Click on the hyperlinks for more details of the test.

Alert level A

```
PLAT721_ALERT_1_A Bond    Calc     0.00000, Rep    3.491(3) Dev...       3.49 Ang.
               CE1      -CE1            1_555   1_555 ........     #    1 Check
```

Author Response: Ce is metallic, this atom separation is correct.

Alert level C

```
PLAT148_ALERT_3_C s.u. on the      a    - Axis is (Too) Large ....      0.005 Ang.
```

Author Response: s.u. is reasonable for trace phase from high pressure data.

Alert level G

```
PLAT004_ALERT_5_G Polymeric Structure Found with Maximum Dimension          2 Info
```

```
  1 ALERT level A = Most likely a serious problem - resolve or explain
  0 ALERT level B = A potentially serious problem, consider carefully
  1 ALERT level C = Check. Ensure it is not caused by an omission or oversight
  1 ALERT level G = General information/check it is not something unexpected

  1 ALERT type 1 CIF construction/syntax error, inconsistent or missing data
  0 ALERT type 2 Indicator that the structure model may be wrong or deficient
  1 ALERT type 3 Indicator that the structure quality may be low
  0 ALERT type 4 Improvement, methodology, query or suggestion
  1 ALERT type 5 Informative message, check
```

It is advisable to attempt to resolve as many as possible of the alerts in all categories. Often the minor alerts point to easily fixed oversights, errors and omissions in your CIF or refinement strategy, so attention to these fine details can be worthwhile. It is up to the individual to critically assess their own results and, if necessary, seek expert advice.

PLATON version of 15/01/2026; check.def file version of 02/01/2026

Datablock Pb - ellipsoid plot

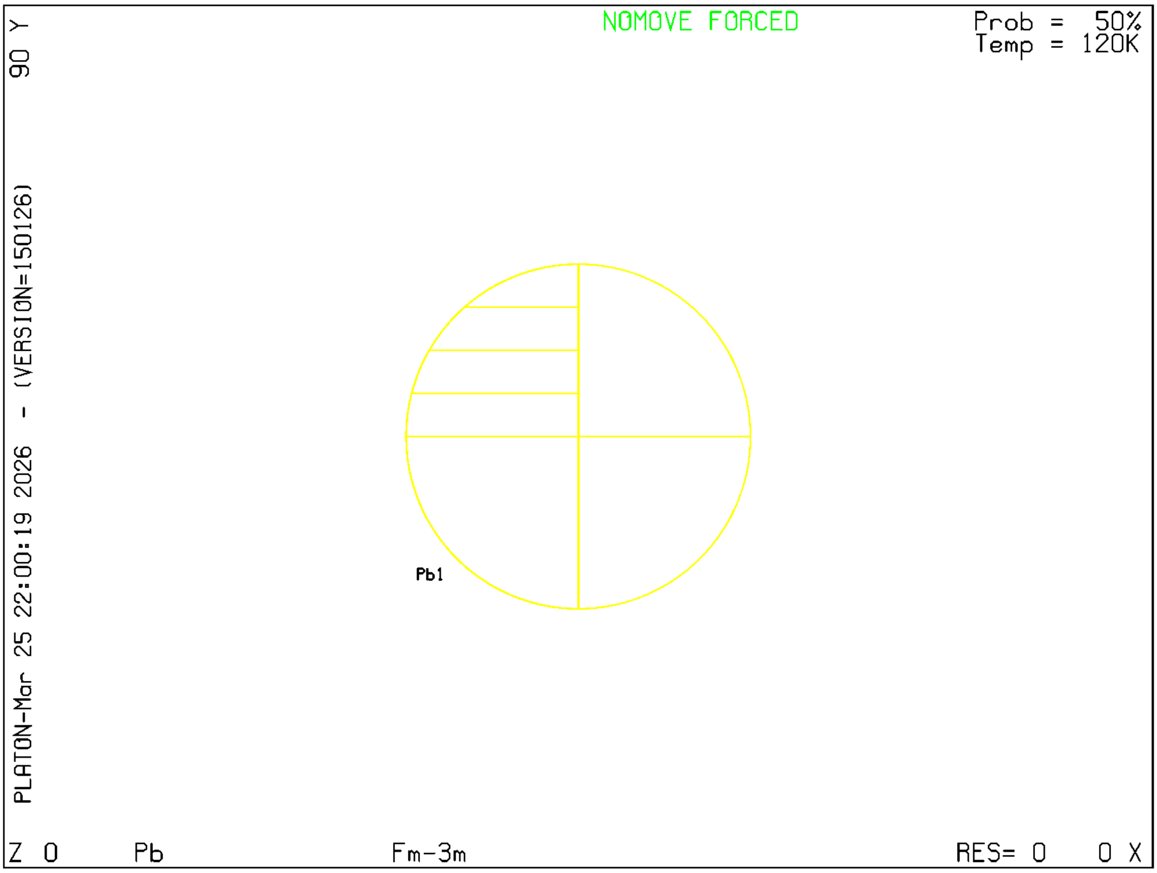

Datablock Cerium_alphaPP - ellipsoid plot

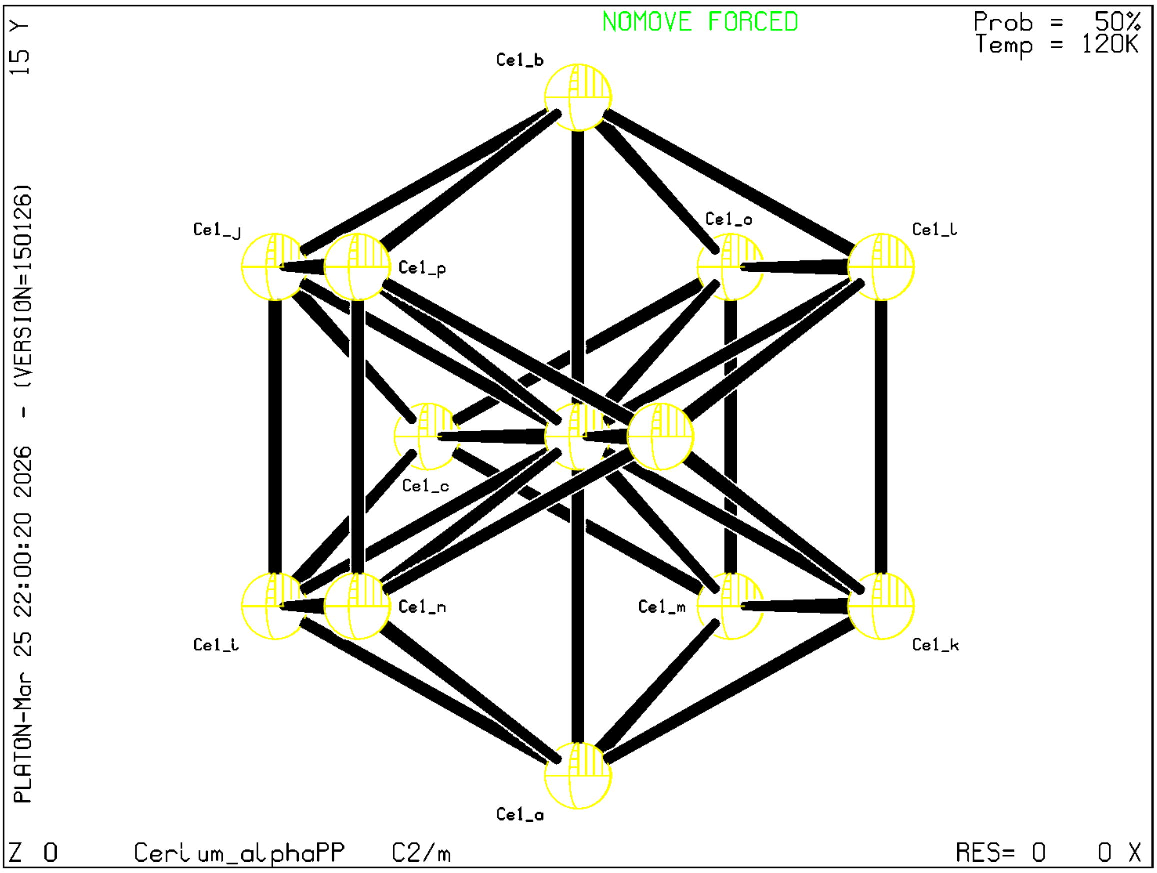

Datablock Steel - ellipsoid plot

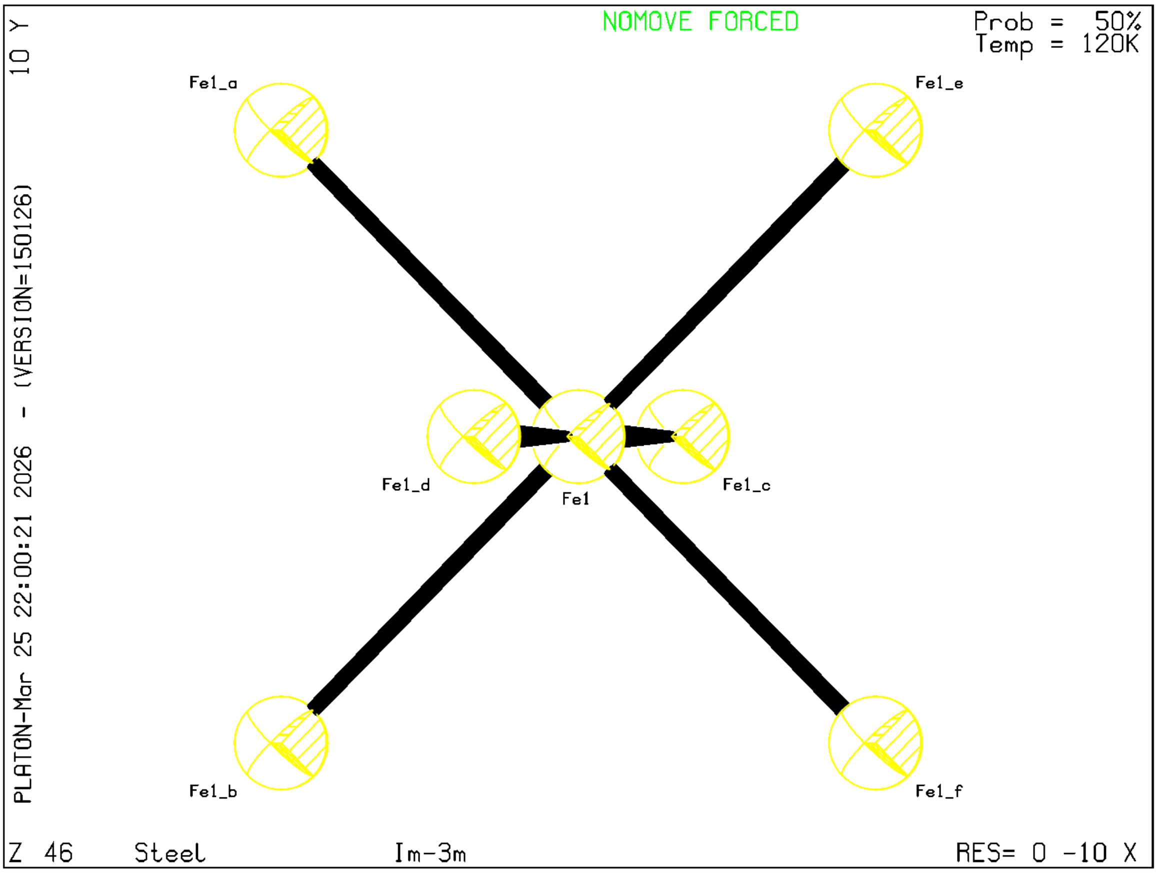

Datablock cBN - ellipsoid plot

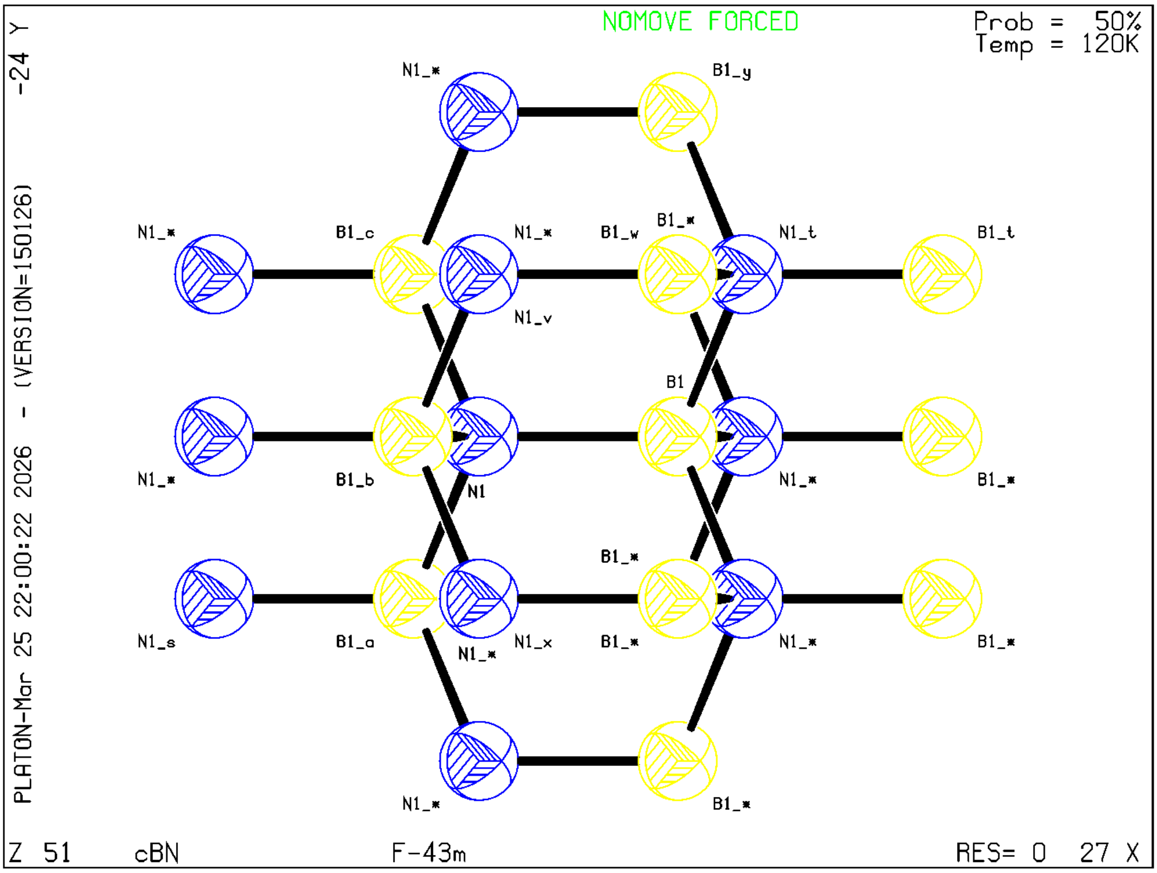

Datablock Cerium_gamma - ellipsoid plot

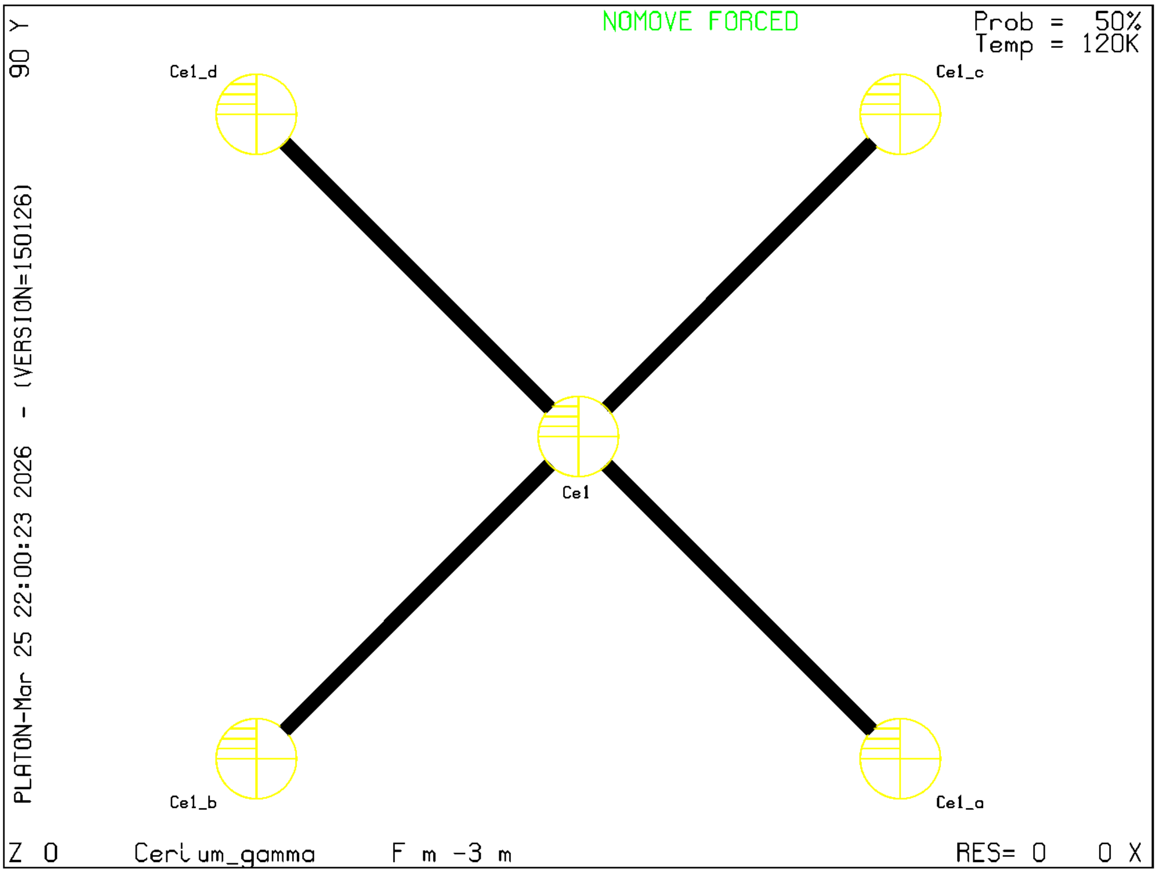

Supplement: Supplementary file 1 — Supplementary Information [file 41467_2026_74329_MOESM1_ESM.pdf]
