# Supplementary material for: The impact of metastability on the high-pressure behavior of cerium
Source: Nat Commun. 2026 Jul 13;17:6133. doi: 10.1038/s41467-026-74329-w (PMC13365817; doi:10.1038/s41467-026-74329-w)
Supplement: Supplementary file 2 — Description of Additional Supplementary Information [file 41467_2026_74329_MOESM2_ESM.pdf]

Description of Additional Supplementary Files

File Name: Supplementary Data 1
Description: isoviz file allowing the reader to animate the symmetry mode relation between the monoclinic and orthorhombic structures of cerium.

File Name: Supplementary Data 2
Description: contains the powder refinement details and refined structures for cerium at 120 K and 6.44(14) GPa (from pellet 3). These have been deposited at the Cambridge Crystallographic Data Centre, under deposition numbers CCDC 2526171 and 2526172.
