# Supplementary material for: The impact of metastability on the high-pressure behavior of cerium
Source: Nat Commun. 2026 Jul 13;17:6133. doi: 10.1038/s41467-026-74329-w (PMC13365817; doi:10.1038/s41467-026-74329-w)
Supplement: Supplementary file 5 — Transparent Peer Review File [file 41467_2026_74329_MOESM5_ESM.pdf]

nature portfolio

Peer Review File

The impact of metastability on the high-pressure behavior of cerium

Corresponding Author: Dr Christopher Ridley

This file contains all reviewer reports in order by version, followed by all author rebuttals in order by version.

Attachments originally included by the reviewers as part of their assessment can be found at the end of this file.

Version 0:

Reviewer comments:

Reviewer #1

(Remarks to the Author)
This manuscript reports on the pressure-temperature studies on the phase transformations in elemental Cerium. The rare earth metal Cerium occupies a unique position in the periodic table due to 4f delocalization/hybridization and resulting volume collapse (FCC to FCC) at high pressures. The pressure-temperature phase diagram of Cerium has also attracted extensive studies during the past several decades on what comes next after the volume collapse in Cerium. There has been considerable debate on the relative stability of orthorhombic (alpha prime phase) and monoclinic (alpha double prime phase). The authors bring fresh perspective to this problem using neutron diffract under high pressures and low temperatures. Neutron diffraction is a bulk technique and does provide new insights into this long-standing controversy in condensed matter physics. The authors have carried out very detailed structural analysis under a variety of temperature and pressure conditions. I recommend this paper for publications provided authors address the following issues.

(1) The issue of negative compressibility reported for the metastable gamma phase requires additional clarification. This is based on the measured d-spacings for various reflections reported in FIG. S1 in the supplementary documentation. It is well known for FCC metals that (200) reflection shows high compressibility due to anisotropic mechanical behavior. It would be instructive to include a similar analysis for the alpha phase that is also FCC to convince readers that negative compressibility is indeed due to metastable gamma phase.

(2) In the proposed phase diagram in Fig. 3, the epsilon phase is not identified. Since the experimental measures have been carried out between 100 K and 295 K, It is premature to extend the phase diagram to OK above 4 GPa.

Reviewer #2

(Remarks to the Author)
Comments and suggestions are provided in the attached .pdf file.

Reviewer #3

(Remarks to the Author)
This paper reports large volume cell studies of cerium metal at high pressure using neutron diffraction. Various P and T paths are followed and conclusions drawn about the metastability (or otherwise) of high pressure phases.

The behaviour of cerium at high pressure is a longstanding problem as is attested by the extensive reference list of this paper. The authors review earlier work and seem to have made a significant contribution through an approach that has not been tried before. The work is competently carried out and presented and I advise the paper should be accepted.

Version 1:

Reviewer comments:

Reviewer #1

(Remarks to the Author)
The authors have addressed all my comments/suggestions including modifications in the phase diagram and including discussion of the anisotropic shifts in the Bragg peak positions.

I am satisfied with the revisions and recommend this manuscript for publication.

Reviewer #2

(Remarks to the Author)
The comments are provided within the attached PDF file.
I consider that the efforts made by authors to improve their manuscript are significant.
I recommend publication of this paper after implementing suggestions and corrections pointed out in the file attached.

Version 2:

Reviewer comments:

Reviewer #2

(Remarks to the Author)
I thank the authors for the careful revision of the manuscript and the associated data, and I recommend the paper for acceptance.

Open Access This Peer Review File is licensed under a Creative Commons Attribution 4.0 International License, which permits use, sharing, adaptation, distribution and reproduction in any medium or format, as long as you give appropriate credit to the original author(s) and the source, provide a link to the Creative Commons license, and indicate if changes were made.
In cases where reviewers are anonymous, credit should be given to 'Anonymous Referee' and the source.
The images or other third party material in this Peer Review File are included in the article's Creative Commons license, unless indicated otherwise in a credit line to the material. If material is not included in the article's Creative Commons license and your intended use is not permitted by statutory regulation or exceeds the permitted use, you will need to obtain permission directly from the copyright holder.
To view a copy of this license, visit https://creativecommons.org/licenses/by/4.0/

We thank the reviewers for their positive and constructive feedback. All changes are detailed below in a point-by-point response and all changes made to the main body of the revised manuscript are marked up in blue.

Reviewer #1:

This manuscript reports on the pressure-temperature studies on the phase transformations in elemental Cerium. The rare earth metal Cerium occupies a unique position in the periodic table due to 4f delocalization/hybridization and resulting volume collapse (FCC to FCC) at high pressures. The pressure-temperature phase diagram of Cerium has also attracted extensive studies during the past several decades on what comes next after the volume collapse in Cerium. There has been considerable debate on the relative stability of orthorhombic (alpha prime phase) and monoclinic (alpha double prime phase). The authors bring fresh perspective to this problem using neutron diffract under high pressures and low temperatures. Neutron diffraction is a bulk technique and does provide new insights into this long-standing controversy in condensed matter physics. The authors have carried out very detailed structural analysis under a variety of temperature and pressure conditions. I recommend this paper for publications provided authors address the following issues.

Response: We thank the reviewer for their appreciation of our new insights, their positive feedback and recommendation to publish. We have addressed their comments as detailed below.

Comment 1: *The issue of negative compressibility reported for the metastable gamma phase requires additional clarification. This is based on the measured d-spacings for various reflections reported in FIG. S1 in the supplementary documentation. It is well known for FCC metals that (200) reflection shows high compressibility due to anisotropic mechanical behavior. It would be instructive to include a similar analysis for the alpha phase that is also FCC to convince readers that negative compressibility is indeed due to metastable gamma phase.*

Response: We have extended this analysis to the alpha phase, which does not show the same hkl-dependent shift (figure S4). We have broadened the discussion around this point in the discussion around the phase stability, extending it with a peak shape analysis of each of the reflections.

> **Line 280:** **“**As the α′→α″ transformation requires only a small shear of the cell together with a secondary Ce displacement, defects capable of storing or accommodating local shear, such as dislocation structures, intervariant boundaries, and possibly planar faulting, may locally favor one distortion state over the other. In our measurements, the anomalous hkl-dependent peak shifts observed for γ-Ce in the low-pressure mixed-phase region at 100 K, but not in the emergent isostructural α-phase, together with the strong texture of α′ formed during slow room-temperature compression and the persistent retention of remnant γ-Ce, are consistent with such a defect-rich inherited microstructure. Fault-related hkl-dependent peak shifts and line-shape changes are well documented in other fcc metals (Velterop et al., 2000; Shih et al., 2021), and their prominence is

known to vary strongly with deformation pathway (Sharma et al., 2020). High stacking-fault probabilities under deformation have been reported for Ce (McHargue, 1961), and there is some evidence for internal strain or stacking faults in Pr under high pressure (Baer et al., 2003). In the present study, the absence of similarly strong hkl-dependent shifts for γ-Ce at room temperature likely reflects the greater ability of the defect structure to relax and redistribute stress during compression, whereas at low temperature the inherited anisotropic microstructure is more effectively retained. In this view, the low-temperature peak shifts are consistent with frozen-in defect-mediated shear accommodation, rather than with a generic property of the fcc lattice itself. We therefore regard stacking faults as a plausible contributor to shear accommodation within the broader microstructural picture of Ce, although we cannot establish them as the unique mechanism selecting between α′ and α″."

Comment 2: *In the proposed phase diagram in Fig. 3, the epsilon phase is not identified. Since the experimental measures have been carried out between 100 K and 295 K, It is premature to extend the phase diagram to OK above 4 GPa.*

Response: The phase diagram has been adjusted, now cropped to ~70 K. Each of the phases is discussed in the introduction, and the caption now includes the space-group assignments of each phase. We have additionally added some further details on past studies of the epsilon phase to highlight that low-temperature compression is expected to push the transition to the epsilon phase to higher pressures, well beyond the pressure regime studied here.

Line 253: While the ε-phase is included in the high-temperature region of the phase diagram following Zhao et al. (1997), its metastable character remains under discussion (Tsiok et al., 2001). At room temperature, ε-Ce has been reported to form upon compression at 12–13 GPa (Gu et al., 1995; Munro et al., 2020; Olsen et al., 1985). However, on a low-temperature compression isotherm, previous X-ray studies reported its onset only at 26 GPa (Olsen et al., 1993), implying a negative slope of the relevant phase boundary. This is consistent with our observations, in which no evidence for ε-Ce was found over the pressure–temperature range investigated.

At room temperature the two high-pressure phases discussed here, α′-Ce and α″-Ce, are energetically similar, …"

Reviewer #2:

Thank you for the opportunity to read and review the manuscript named: "The impact of metastability on the high-pressure behavior of cerium".

At the moment of writing, I have mixed feelings and would recommend a major revision of the manuscript. There are several disturbing observations, some of them are minor, some of them are of considerable importance and should be fixed.

Below, I provide a list of comments and suggestions that should help improve the manuscript before it can be considered for acceptance.

Response: We thank the reviewer for their careful reading, and insightful suggestions. We have addressed the concerns as detailed below.

Comment 1: *Line 26 (Abstract): "slow compression at room temperature favors an 26 orthorhombic phase (α), whereas compression at low temperature stabilizes a pure monoclinic phase (α'')"*

Please make the text more consistent: first of the sentence add kinetics of compression suggesting its importance, while the second part of the sentence avoids any compression speed comparison.

Response: We have clarified the sentence to:

> **Abstract, line 25:** "slow compression at room temperature favors an orthorhombic phase (α′), whereas slow and also moderately faster compression at low temperature stabilizes a pure monoclinic phase (α′′)."

Comment 2: *Line 113 (Compression At 295 K): "The possibility of this instead being an impurity phase emergent at high pressures has been eliminated (see SI)."*

Please indicate here which phases were considered for assessment and elimination. I also suggest a modification: "The possibility that this is instead ..".

Response: We have included this in the main text as follows:

> **Line 113:** "The possibility that this is instead one of the typical impurity phases, namely CeO, CeO2, or CeH2, emergent at high pressures, or PbO from the pressure marker is discussed more extensively in the SI, and ultimately eliminated."

Comment 3: *Line 123 (Compression At 295 K): "The persistence of the trace γ phase through the transition is curious"*

The authors discuss here the influence of grain size of a pure phase, but omit here and below the important factors of defect density coupled with the original grain size (mortar was used here?) and the potential contribution of Pb (is there evidence suggesting that we can neglect its contribution?). It requires expanded discussion either in the main text or supplementary.

Response: We have expanded the discussion to clarify that grain size is not an independent variable in our samples, but is coupled to defect density, residual strain, and texture through the preparation route. The text now refers more broadly to the retained microstructural state as the controlling factor in phase selection. We also clarify the role of Pb as an *in situ* pressure marker and note that, while a secondary mechanical contribution cannot be excluded, it is unlikely to be the primary driver of the observed behaviour.

> **Line 134:** "In the present samples, the starting point was a pre-pressed pellet of coarse Ce powder and Pb foil, such that particle size, cold-work defect density, residual strain, and texture are inherently coupled. The observed **α'/α"** selection is therefore more appropriately attributed to the retained microstructural state rather than grain size alone. Pb was used as an *in situ* pressure marker; while a secondary mechanical role in redistributing local stress cannot be excluded, the strong dependence of phase selection on pressure–temperature pathway across identically prepared pellets indicates that retained Ce microstructure, rather than Pb content itself, is the dominant factor. Following the work of Henry et al., it is therefore extremely likely that the amount of remnant **γ** phase present affects this process. "
>
> **Methods, line 353:** "This translates to a mass ratio for Ce:Pb of 32:7 and a volume ratio of 31:4. The materials were gently mixed (but not ground) in the mortar, whereby the Pb foils remained as solid pieces throughout."

Comment 4: *Fig 2 (Compression At 295 K) + Methods:*

I would like to ask the authors to make the pictures consistent. Please indicate in (b) where one could expect α' phase, since the number of phases and lines is large and it is already hard for an expert, meaning that it will be impossible for a general reader to see it. The Methods section indicates that Rietveld analysis was applied. Was it applied to all the phases indicated? Or some of the phase contributions were simulated using Pawley/Le-Bail approaches? This is important and should be stated clearly. These nuances could contribute, on various levels, including the phases stability field or relative phase abundance misinterpretation. For example, the Fig. 2b carries quite a bit of Pb and Fe signal overlapped at lower d-spacing with the signal of α' or α"phases.

Response: The fits are indeed full Rietveld (all the phases included use the full structural model for profile calculation). This has now been emphasised in the methods section:

> **Line 368:** "Full Rietveld refinements (for all included phases)"

The figures are consistent, in that only the phases present in each of the fit are included as 'tick'-marks at the bottom. We have made an additional SI figure (Figure S9), which shows a decomposed version of this fit, also indicating where one would expect to see the alpha-prime phase, if it were present.

Comment 5: *Lines 134-151 (Compression At 295 K):*

I suggest that an improvement should be made for this paragraph. For comparison with the other studies, it should be mentioned that studies are done using different high-pressure environments. For example, McMahon and Nelmes [9, 10] use diamond anvil cells, thus, their sample amount is substantially different, not to mention different pressure media used, potentially much more hydrostatic than Pb and or Ce itself. This discussion is clearly missing, but it would confuse the general reader. There is a brief table in supplementary, but this is not sufficient, as the discussion in the main text is formed in such way that the experiments [9] and [10] are similar to those of Paris-Edinburgh press, although they are quite different.

I would suggest that the current study does not "contradict" the result of McMahon, but, provides another perspective with larger sample volume and potentially different grain averaging. With respect to Refs. [9, 10] it is of slightly confusing, how one would approach them in terms of reproducibility and sample uniqueness, it is somewhat example of cut and paste text, especially in their methods description. If the beamsize is ~75 um (FWHM, similar to doi: 10.1103/PhysRevB.51.15723), one could think about grain size of [10], given full rings coverage at the detector side. This discussion could enrich the Supplementary.

If I understand the method used in the publication correctly, in addition to general discussion, one could apply peak width analysis to the data (especially those patterns collected at ambient or low pressure), trying to look at the initial strain and grain size (ideally providing some upper and lower bounds). Would it be possible to add this data to strengthen the publication? It will significantly improve the discussion and it will also provide a point of reference for future studies.

Response: We have expanded the section around the table mentioned by the reviewer (Tab. S2) in the SI to include a significantly more detailed discussion on comparability of hydrostaticity, sample purity and sample volume. We have additionally modified the text in question to better clarify the differences:

> **Line 146:** "Our measurements expand on this finding. Using toroidal anvils with their cupped recession for sample space allows many magnitudes of order larger sample volume coupled with lower levels of deviatoric stresses (see SI for details). Now even when starting with a coarse powder, akin to filings, subsequently cold-worked into a dense pellet prior to further pressurization, a majority α′ forms with a trace of α′′, an outcome previously only observed from

> ‘cut-pieces’ (100-200 μm across). In this context, diffraction studies probing only small sample volumes may misrepresent phase fractions in a strain-heterogeneous material, whereas large-volume neutron diffraction provides a more representative measure of the phase coexistence.”

We have followed the reviewers suggestion and have included peak-width analysis over the low pressure region considered for the gamma phase, accompanying the hkl-dependent peak shifts. It is not possible to extend this over a broader pressure range, due to large amounts of peak overlap. This has been used in a broader discussion relating to changes in the microstructure and the potential influence of stacking faults:

> **Line 280: “**As the α′→α″ transformation requires only a small shear of the cell together with a secondary Ce displacement, defects capable of storing or accommodating local shear, such as dislocation structures, intervariant boundaries, and possibly planar faulting, may locally favor one distortion state over the other. In our measurements, the anomalous hkl-dependent peak shifts observed for γ-Ce in the low-pressure mixed-phase region at 100 K, but not in the emergent isostructural α-phase, together with the strong texture of α′ formed during slow room-temperature compression and the persistent retention of remnant γ-Ce, are consistent with such a defect-rich inherited microstructure. Fault-related hkl-dependent peak shifts and line-shape changes are well documented in other fcc metals (Velterop et al., 2000; Shih et al., 2021), and their prominence is known to vary strongly with deformation pathway (Sharma et al., 2020). High stacking-fault probabilities under deformation have been reported for Ce (McHargue, 1961), and there is some evidence for internal strain or stacking faults in Pr under high pressure (Baer et al., 2003). In the present study, the absence of similarly strong hkl-dependent shifts for γ-Ce at room temperature likely reflects the greater ability of the defect structure to relax and redistribute stress during compression, whereas at low temperature the inherited anisotropic microstructure is more effectively retained. In this view, the low-temperature peak shifts are consistent with frozen-in defect-mediated shear accommodation, rather than with a generic property of the fcc lattice itself. We therefore regard stacking faults as a plausible contributor to shear accommodation within the broader microstructural picture of Ce, although we cannot establish them as the unique mechanism selecting between α′ and α″.”

Comment 6: *Line 150 pressure annealing*

Here and below, I strongly recommend on a revision of wording. Please consider using the terms, “pressure stabilization”, “thermodynamic equilibration”. The grain growth of the transformed phase in this case differs from thermally driven processes such as annealing, where grains can grow and eliminate dislocation defects. During a solid-phase transformation, however, as kinetics is suppressed, grain growth is constrained by the existing defect network. As a result, the newly formed grains have little opportunity to fully reorganize this network and instead tend to adapt to it.

Yes, this is a phase transition driven by a complex energy landscape (defects, microstrains, potentially non-homogeneous stress distribution, and inter-grain contact). Some degree of metastability may be involved, but it is certainly not an annealing process.

Response: The text has been adjusted to reflect this and we have adopted the phrase "pressure cycling" used by Zachariasen and Ellinger throughout, as shown in the example below:

> **Line 159:** "Zachariasen and Ellinger [31] observed a mixture of α′ and α″ consistently, with no measurable change in a sample held at pressure for 10 days, but pressure cycling shown to encourage the formation of α′.

Comment 7: *Line 154 (low temperature compression):*

γ (85%) & β (15%) - For this case and similar, the error bars have to be provided.

Compression to ~0.07 GPa

It is important to address the topic of the accuracy and precision of Pb equation of state used and how it is translated to the error bars of pressure determination. Please indicate the errorbars for all pressures shown within the manuscript. These errorbars can be calculated via the corresponding values (unit cell volume, bulk modulus at ambient pressure, its pressure derivative as well as their corresponding error bars for the reported Pb equation of state).

Response: These have now been included throughout, where specific values are referred to. Additionally, more detail on the Pb equation of state used here has been provided in the Methods section:

> **Methods, line 371:** "The pressure was extracted from the Pb pressure marker using a parameterization of literature data for a pressure- and temperature-dependent equation of state of the face-centered cubic phase of Pb [72]."

Comment 8: *Lines 153-165 (low temperature compression), including: "On close inspection of the Rietveld fits over this pressure region, there is a strong hkl -dependent peak offset, which could be indicative of non-hydrostatic conditions (see SI). However, the low bulk modulus of Ce makes this explanation unlikely."*

The argument of a low bulk modulus cannot be applied here to a large extent. Although the compressibility is high, this does not mean that effects of non-hydrostatic stresses and microstrains (between Ce grains and Ce-Pb grains), leading to peak shifts, cannot occur in this soft phase. While such effects may complicate the analysis, strain-related peak shifts should be treated carefully and, if possible, analyzed on a peak-by-peak basis. If a reliable Rietveld model that properly accounts for peak shifts

cannot be constructed, the analysis can no longer be considered a true Rietveld refinement. Neglecting these effects would make the results more ambiguous. It should also be noted that peak shifts relative to the ideal positions may indicate a phase transition; therefore, this observation should be treated with particular care. One could also consider of addressing the magnitude of these shift, if it is considerable or minor.

This is an experiment involving Paris-Edinburgh press at low temperature conditions without a quasihydrostatic pressure medium, so one should expect presence of non-hydrostatic strains and stresses. It would be suggested, that both – non hydrostaticity and γ phase metastability be considered as major contributions. One potentially cannot choose one over the other, since strains complicate local energy landscapes and can indeed promote metastability (like for example in doi: 10.1103/PhysRevB.100.064104)

Response: To get a better insight into hydrostaticity levels across the various studies, a more detailed discussion has been added to the SI, Section III. This has highlighted that the particular conditions of the cupped sample space used in toroidal anvils together with the cell/beam geometry should yield lower deviatoric stresses than the majority of diamond cell experiments. The sentence on the low bulk modulus has been removed and replaced as follows. Additionally, the methods section has been updated to specifically include the cell/beam geometry.

> **Line 171:** "On close inspection of the Rietveld fits over this pressure region, there is a strong hkl-dependent peak offset, which could be indicative of non-hydrostatic conditions (see SI). However, the particular set-up used here consisting of anvils with cupped recessions for sample and beam-through-gasket geometries are expected to observe low deviatoric stresses only (see SI). We thus suggest it is likely related to the metastability of γ at this temperature, kinetically trapped through fast cooling."
>
> **Methods, line 336:** "For all experiments, the cells were placed in a geometry so that beam enters and scatters through the gasket. 3D-printed B4C slits were used to shape the incident beam to minimize parasitic scatter [64]."

Comment 9: *Lines 164-168 (low temperature compression),: "The sample was then heated to 190 K, removing all trace of β, and cooled back to 100 K, yielding a mixture of α with a small trace of γ. Above 5.2 GPa additional peaks appear in the pattern which can only be fully accounted for by monoclinic α'."*

Please clarify at which temperature the data collection was made for 5.2 GPa, otherwise the looks discussion is slightly ambiguous.

Response: This has now been updated to clarify that all the compression occurred at 100 K.

> **Line 178:** "At a constant load of 26.7 metric tons, the sample was then heated to 190 K, removing all trace of β, and cooled back to 100 K, yielding a mixture of α with a small trace of γ. Thereafter, compression continued at a constant 100 K."

Comment 10: *Fig. 3 + text (towards a phase diagram)*

Please provide indication of corresponding space groups for each phase either within the phase diagram or in a legend – this will improve a comprehension. One of the important questions which remains unanswered in this part is a crystallographic relation between different phases, in particular the α' and α". Could the authors indicate more clearly for general audience if they are really so different or if they can be constructed from one another in an easy way? Could there be a process and a relevant interface making, for example a simple stack fault keeping one phase stabilized over or together with the other? Could there be a group-subgroup relation to be discussed?

Response: The space-group assignments of each phase have now been included in the caption to the figure, for clarity. There is indeed a group-subgroup relation between the alpha' and alpha" phases; we now discuss this and the potential effects of stacking faults in the manuscript mentioned in response to comment 5.

> **Line 269:** "Crystallographically, α′ and α″ are closely related through a group–subgroup symmetry relationship. The α″ (C2/m) structure can be generated from α′ (Cmcm) by a monoclinic shear of the orthorhombic cell together with a secondary displacement of the Ce atoms (see attached supplementary file). In this sense, α″ may be viewed as a symmetry-lowered distorted form of α′, rather than a reconstructive rearrangement. This is consistent with the small volume difference between the two phases and with the ease with which modest heating or pressure cycling seeds α′ and α″. Prior work likewise argued that the orthorhombic and monoclinic forms of Ce are linked by a common distortion mechanism and that their relative stability is highly sensitive to elastic fields and sample preparation (Dmitriev et al., 2004). This close crystallographic relationship also suggests a natural route by which retained microstructure may influence phase selection."

Comment 11: *Conclusions*

While in general the paragraph seems to look valid, it does require a revision. One of my comments is related to comparison of the current work to Ref. [9] conducted using diamond anvil cells. Judging from [9,10], one can come to opposite conclusion – it was different preparation, pressure medium and sample morphology (actually, we do not know much about sample morphology, as there are not pictures available from sample chamber of those publications).

Response: Detailed photos of all cell loadings and sample materials is difficult to quantitatively compare between experiments. Further, the differences in the diamond cell work [9] and our work has been much better addressed throughout the paper now. The beginning of the conclusion has now been reworked to reflect these points.

Line 306: We show that under room-temperature compression in a large-volume cell with reasonable hydrostaticity ultra-high-purity Ce transforms into a highly textured orthorhombic α′ (α-U) structure with a small coexisting α″ fraction. This is a surprising finding for a sample formed by cold working a coarse powder such as ours since previous observations on samples with similar preparation and morphology [9] yielded pure α″.

Comment 12.1: *Additional, but very comments regarding the supplementary files, Fig. S3-S5*

Please indicate tick marks of the impurity phases of Pb, Fe and cBN, at least at the bottom pattern for each Figure.

It is surprising that the noise within the patterns allowed you to extract up to 399 independent peaks (powder Rietveld refinement) for one of the phase of the provided CIFs. Could some of those be due to the background noise? The signal to noise ratio is not too inspiring if one looks at d-spacings of 0.8-1.2 Å – there is large scatter.

Why there is such a strange variation of intensities in Fig. S3 between the low pressure patterns below 0.73 GPa, and those above 0.73 GPa?

Similar question can be applied for Fig. S5, but in the opposite – lower pressures seem to have more sample intensity than the higher one.

Response: The placement of tick marks in these stack plots is likely to cause confusion, as the peaks move past each other with pressure in some cases (Pb) but not others (cBN). Instead, we have included a new figure in the SI (Figure S9) showing a decomposed Rietveld for figure 2b, which makes it clear which peaks belong to which phase.

The number of refined reflections was incorrectly reported in the cif. These were reported erroneously, as data were excluded below a d-spacing cut-off. This has now been corrected, and the total number of reflections (from all the phases together) is closer to 200, well within the manageable range of Rietveld.

The variation in total intensity in the fig S3 and S5 is due to a repositions of the collimator slit between these measurements. As the sample goes through a large volume collapse, the gasket thins significantly. This closes the gap between the anvils, both reducing the level of scattered signal, and requiring that the slit be repositioned. The pressure labels were in fact the wrong way around on this figure, and this has now been corrected.

Comment 12.2: *Issues with the CIF file data*

CIF file – the pressure given within the field _cell_measurement_pressure should be provided in kPa units (https://www.iucr.org/__data/iucr/cifdic_html/1/cif_core.dic/Icell_measurement_pressure.html)

CIF file – _refine_ls_R_I_factor

If I understand correctly, this value should correspond to R1 factor definition similar to the Ref. (https://www.iucr.org/__data/iucr/cifdic_html/1/cif_core.dic/Irefine_ls_R_I_factor.html).

The values of 2.06456 and 1.63519 provided in CIF should correspond, according to the definition, to R1 of ~207 % and 163 %, which is too large. It should be discussed or explained. It may be a software issue, but it should be addressed.

In the prepared CIF I could not find the number of refined parameters, residual electronic density if the authors indeed used Rietveld refinement which should indicate the quality of the data analysis.

CIF file ***– It is a major issue with sigma values for intensity****. Indeed σF() it is much larger than the intensity itself. Here is an indicative and self-explanatory snapshot from the supplementary CIF file showing several fields, including the reported standard deviations of the intensities. These may be related to software processing, but they do not present the data in a favorable light, potentially an oversight.*

Response: The pressure units have been corrected. The R-factors reported have also been labelled appropriately, showing that the fit is very good. The number of refined parameters is listed in the cif. The reported sigma values were incorrect due to an error in the refinement script. In line with guidelines for powder-cifs, we have updated the format to include all the fitted phases, and the hkl_yobs-ycalc for all the phases included in the pattern. In powder diffraction, individual reflection intensities are not directly measured but are obtained from a global profile (Rietveld) fit to overlapping peaks. As a result, F^2 values are model-derived and do not have independently meaningful uncertainties, so reporting sigma(F^2) is not standard practice. So while we have noted that these values were incorrect, we have removed these values and instead provide the profile information in line with conventional powder CIF reporting.

Reviewer #3:

This paper reports large volume cell studies of cerium metal at high pressure using neutron diffraction. Various P and T paths are followed and conclusions drawn about the metastability (or otherwise) of high pressure phases.

The behaviour of cerium at high pressure is a longstanding problem as is attested by the extensive reference list of this paper. The authors review earlier work and seem to have made a significant contribution through an approach that has not been tried before. The work is competently carried out and presented and I advise the paper should be accepted.

Response: We thank the reviewer for their positive feedback, their appreciation of the novelty of our approach, and their recommendation to accept our manuscript.

Response to reviewer #2: please find our response below in **blue**.

Abstract Line 26:

To make the discussion more quantitative, I would greatly appreciate it if the authors could indicate the maximum strain rate (for example, <XXX or YYY) used to characterize the moderately faster compression. Given the increasing relevance of dynamic compression, it is important to provide at least some basis for comparison.

The rates of pressure increase have now been included.

"We find that the crystal structure formed at high pressure depends on the compression pathway: slow compression (~0.25 GPa hr^{-1}) at room temperature favors an orthorhombic phase (α'), whereas both slow (~0.25 GPa hr^{-1}) and moderately faster (~0.5 GPa hr^{-1}) compression at low temperature stabilize a pure monoclinic phase (α'')."

Figure 1b

The d-spacing range 2.5–2.6 should be clearly indicated. It should be made explicit where 2.5 starts and where 2.6 ends (for example, left/right or top/bottom). This may be clear to a scientist who analyzed the data, but it will not necessarily be clear to a general reader.

Time-of-flight neutron diffraction is a white-beam technique, as such the entire detector represents this d-spacing range, showing the clear texture in the sample forming 'broad single crystal like' reflections. This is now stated in the main text for readers unfamiliar with the technique.

"The sample is illuminated with a white beam of neutrons, with each energy distinguished by its different time-of-flight from the source to the detector. Figure 1b is filtered to show only the neutron signal in the 2.5-2.65 Å d-spacing range, displayed across the entire detector face. It shows single-crystal-like features that reflect the strong texture in the (002) and (021) reflections of α', which are the only reflections present in this d-spacing range."

Lines 148 and 175

Please revise:
"coupled with lower levels of deviatoric stresses"
to:
"coupled with potentially lower levels of deviatoric stresses"

This has been altered as requested.

The statement that low deviatoric stresses are expected to be observed (see SI) is not yet sufficiently supported. The Supplementary Information only discusses flat and recessed anvils. While this is a useful discussion, it is not sufficient on its own. Many factors should be considered, including temperature. Helium is a good quasihydrostatic pressure medium when liquid, but is it still quasihydrostatic at low temperatures when it is solid? More generally, what is more quasihydrostatic at low temperatures: liquid He or solid metal Ce?

I suggest that, in a brief surge of enthusiasm, the authors made several attempts to offer a simple, but less scientific, explanation of a difficult challenge, perhaps with the intention of leading a general reader to a more positive impression.

For an experienced group working under high pressure, it cannot simply be “expected” that low deviatoric stress will be observed. Either there is experimental evidence for this, or there is not.

We have made some edits to this passage to reflect some of the concerns of the reviewer, however we believe that the levels of deviatoric stress in the sample are well quantified through the measurements reported in the Klotz reference [50], and that we suitably motivate our favoring alternative reasons for the observed peak shifts. In particular the fact that no such effect is seen at much higher pressures at the same temperature for the alpha phase (Fig S4).

“It is understood that deviatoric stresses can develop under high-pressure conditions when no pressure medium is present, and that these effects may be exacerbated at low temperatures. The pressure assembly used here, consisting of toroidal anvils with cupped recesses for the sample, typically generates relatively low levels of deviatoric stress, as demonstrated at room temperature using a test sample of NaCl [50], which has a similar bulk and shear modulus to Ce. The scattering geometry used in the present study samples only a narrow plane through the center of the sample-gasket cross-section, where the lowest levels of deviatoric stress were observed (discussed further in the SI). While the potential effects of these stresses cannot be ruled out entirely, we suggest that our observation is more likely related to the metastability of γ at this temperature, kinetically trapped through fast cooling.”

Lines 249–252

“Under low-temperature compression, identically prepared pellets consistently transform into purely monoclinic α′′-Ce, with no detectable α′-Ce. In contrast, room-temperature compression produces a highly textured orthorhombic α′-Ce phase with a small coexisting fraction of α′′-Ce.”

I suggest revision. This text compares low-temperature compression with ambient-temperature compression conducted by the same group. Under room-temperature compression, a highly textured α′-Ce phase is produced. However, the use of “in contrast” may give the impression that the sample obtained under lower-temperature compression is not highly textured, which may not be the case.

This passage also contributes to the overall discussion of hydrostaticity and suggests that the authors should provide additional information on how texture and preferred orientation effects were treated in the full Rietveld refinements. At present, this important information is missing from both the Methods section and the provided data.

That is correct. The texture is absent in the low temperature compressions. This is now spelled out in the main text.

“The low-temperature monoclinic phase shows no such texture.”

The texture of the orthorhombic phase is treated using preferred orientation modelling. This is now clearly stated in the Methods section.

“The strong texture, observed only in the orthorhombic phase during the room temperature compression, was accounted for using preferred orientation modeling in Topas v6. To avoid issues with correlation in the refinements, the Ce coordinate in this phase was fixed.”

Supplementary Information, Table S2:
The McMahon [10] reference is cited in the SI as using no PTM, but the original paper clearly states that McMahon et al. also attempted methanol-ethanol PTM studies. This appears to have been overlooked, although it is relevant to the discussion of hydrostaticity.

This has been updated in the table.

Comments on the replies from the first iteration

However, I would also like to better understand the relatively high goodness-of-fit value in comparison with the R-factor values, which appear fairly good and low:

_refine_ls_goodness_of_fit_all 2.49519
_pd_proc_ls_prof_R_factor 0.01639
_pd_proc_ls_prof_wR_factor 0.02064

Since the previous version of the most important piece of evidence—the submitted CIF file—had severe issues, I would like the authors to double-check and explain whether this value is valid, and if so, what the high value might be related to. A value of 2.5 is rather high for conventional experiments, and I am not sure whether such values are typical for Paris–Edinburgh experiments.

We welcome the rigorous dissemination of the cif file. The reported cif is indeed now correct, and the goodness of fit is very typical of that expected for the PE-press on the instrument, this is confirmed through measurement of the Si pellet in the press as part of the calibration process. Fitting metrics can be weighted by high backgrounds, and it is considerably more important that the fit is visually good, which is clearly the case in this instance. We have added this point to the data availability statement where the published cif is advertised.

"The reported goodness of fit for this powder-cif is typical of that expected for data collected on the instrument in the Paris-Edinburgh press."

Figure 1b:
Nature Communications policy appears to require scales for all heatmaps.

A heatmap scale bar has been added to Fig1b as requested.

In addition, because the submission does not include raw 1D data and only includes figures, I would like to note that, to the best of my understanding, Nature Communications policy strongly encourages, if not requires, submission of all raw data underlying the figures in both the main manuscript and the Supplementary Information, either alongside the submission or via an online repository such as Zenodo.

The raw data are indeed already available through the published DOI, see [75].

Thank you for the opportunity to read and review the manuscript named: “The impact of metastability on the high-pressure behavior of cerium”.

At the moment of writing, I have mixed feelings and would recommend a major revision of the manuscript. There are several disturbing observations, some of them are minor, some of them are of considerable importance and should be fixed.

Below, I provide a list of comments and suggestions that should help improve the manuscript before it can be considered for acceptance.

1. Line 26 (Abstract): *<u>slow compression</u> at room temperature favors an 26 orthorhombic phase (α), whereas <u>compression</u> at low temperature stabilizes a pure monoclinic phase (α'')*

Please make the text more consistent: first of the sentence add kinetics of compression suggesting its importance, while the second part of the sentence avoids any compression speed comparison.

2. Line 113 (Compression At 295 K): *The possibility of this instead being an impurity phase emergent at high pressures has been eliminated (see SI).*

Please indicate here which phases were considered for assessment and elimination. I also suggest a modification: “The possibility that this is instead ..”.

3. Line 123 (Compression At 295 K): *The persistence of the trace γ phase through the transition is curious*

The authors discuss here the influence of grain size of a pure phase, but omit here and below the important factors of defect density coupled with the original grain size (mortar was used here?) and the potential contribution of Pb (is there evidence suggesting that we can neglect its contribution?). It requires expanded discussion either in the main text or supplementary.

4. Fig 2 (Compression At 295 K) + Methods:

I would like to ask the authors to make the pictures consistent. Please indicate in (b) where one could expect α’ phase, since the number of phases and lines is large and it is already hard for an expert, meaning that it will be impossible for a general reader to see it.

The Methods section indicates that Rietveld analysis was applied. Was it applied to all the phases indicated? Or some of the phase contributions were simulated using Pawley/Le-Bail approaches? This is important and should be stated clearly. These nuances could contribute, on various levels, including the phases stability field or relative phase abundance misinterpretation. For example, the Fig. 2b carries quite a bit of Pb and Fe signal overlapped at lower d-spacing with the signal of α’ or α’’ phases.

5. Lines 134-151 (Compression At 295 K):

I suggest that an improvement should be made for this paragraph.

For comparison with the other studies, it should be mentioned that studies are done using different high-pressure environments. For example, McMahon and Nelmes [9, 10] use diamond anvil cells, thus, their sample amount is substantially different, not to mention different pressure media used, potentially much more hydrostatic than Pb and or Ce itself. This discussion is clearly missing, but it

would confuse the general reader. There is a brief table in supplementary, but this is not sufficient, as the discussion in the main text is formed in such way that the experiments [9] and [10] are similar to those of Paris-Edinburgh press, although they are quite different.

I would suggest that the current study does not “contradict” the result of McMahon, but, provides another perspective with larger sample volume and potentially different grain averaging. With respect to Refs. [9, 10] it is of slightly confusing, how one would approach them in terms of reproducibility and sample uniqueness, it is somewhat example of cut and paste text, especially in their methods description. If the beamsize is ~75 um (FWHM, similar to doi: 10.1103/PhysRevB.51.15723), one could think about grain size of [10], given full rings coverage at the detector side. This discussion could enrich the Supplementary.

If I understand the method used in the publication correctly, in addition to general discussion, one could apply peak width analysis to the data (especially those patterns collected at ambient or low pressure), trying to look at the initial strain and grain size (ideally providing some upper and lower bounds). Would it be possible to add this data to strengthen the publication? It will significantly improve the discussion and it will also provide a point of reference for future studies.

6. Line 150 *pressure annealing*

Here and below, I strongly recommend on a revision of wording. Please consider using the terms, “pressure stabilization”, “thermodynamic equilibration”. The grain growth of the transformed phase in this case differs from thermally driven processes such as annealing, where grains can grow and eliminate dislocation defects. During a solid-phase transformation, however, as kinetics is suppressed, grain growth is constrained by the existing defect network. As a result, the newly formed grains have little opportunity to fully reorganize this network and instead tend to adapt to it.

Yes, this is a phase transition driven by a complex energy landscape (defects, microstrains, potentially non-homogeneous stress distribution, and inter-grain contact). Some degree of metastability may be involved, but it is certainly not an annealing process.

7. Line 154 (low temperature compression):

γ (85%) & β (15%)

For this case and similar, the error bars have to be provided.

Compression to ~0.07 GPa

It is important to address the topic of the accuracy and precision of Pb equation of state used and how it is translated to the error bars of pressure determination. Please indicate the errorbars for all pressures shown within the manuscript. These errorbars can be calculated via the corresponding values (unit cell volume, bulk modulus at ambient pressure, its pressure derivative as well as their corresponding error bars for the reported Pb equation of state).

8. Lines 153-165 (low temperature compression), including: *On close inspection of the Rietveld fits over this pressure region, there is a strong hkl -dependent peak offset, which could be indicative of non-hydrostatic conditions (see SI). However, the low bulk modulus of Ce makes this explanation unlikely.*

The argument of a low bulk modulus cannot be applied here to a large extent. Although the compressibility is high, this does not mean that effects of non-hydrostatic stresses and microstrains (between Ce grains and Ce-Pb grains), leading to peak shifts, cannot occur in this soft phase. While such effects may complicate the analysis, strain-related peak shifts should be treated carefully and, if possible, analyzed on a peak-by-peak basis. If a reliable Rietveld model that properly accounts for peak shifts cannot be constructed, the analysis can no longer be considered a true Rietveld refinement. Neglecting these effects would make the results more ambiguous. It should also be noted that peak shifts relative to the ideal positions may indicate a phase transition; therefore, this observation should be treated with particular care. One could also consider of addressing the magnitude of these shift, if it is considerable or minor.

This is an experiment involving Paris-Edinburgh press at low temperature conditions without a quasihydrostatic pressure medium, so one should expect presence of non-hydrostatic stains and stresses. It would be suggested, that both – non hydrostaticity and γ phase metastability be considered as major contributions. One potentially cannot choose one over the other, since strains complicate local energy landscapes and can indeed promote metastability (like for example in doi: 10.1103/PhysRevB.100.064104).

9. Lines 164-168 (low temperature compression),: *The sample was then heated to 190 K, removing all trace of β, and cooled back to 100 K, yielding a mixture of α with a small trace of γ.*
Above 5.2 GPa additional peaks appear in the pattern which can only be fully accounted for by monoclinic α′.

Please clarify at which temperature the data collection was made for 5.2 GPa, otherwise the looks discussion is slightly ambiguous.

10. Fig. 3 + text (towards a phase diagram)

Please provide indication of corresponding space groups for each phase either within the phase diagram or in a legend – this will improve a comprehension. One of the important questions which remains unanswered in this part is a crystallographic relation between different phases, in particular the α′ and α′′. Could the authors indicate more clearly for general audience if they are really so different or if they can be constructed from one another in an easy way? Could there be a process and a relevant interface making, for example a simple stack fault keeping one phase stabilized over or together with the other? Could there be a group-subgroup relation to be discussed?

11. Conclusions

While in general the paragraph seems to look valid, it does require a revision. One of my comments is related to comparison of the current work to Ref. [9] conducted using diamond anvil cells. Judging from [9,10], one can come to opposite conclusion – it was different preparation, pressure medium and sample morphology (actually, we do not know much about sample morphology, as there are not pictures available from sample chamber of those publications).

12. Additional, but very comments regarding the supplementary files:
12.1 Fig. S3-S5

Please indicate tick marks of the impurity phases of Pb, Fe and cBN, at least at the bottom pattern for each Figure.

It is surprising that the noise within the patterns allowed you to extract up to 399 independent peaks (powder Rietveld refinement) for one of the phase of the provided CIFs. Could some of those be due to the background noise? The signal to noise ratio is not too inspiring if one looks at d-spacings of 0.8-1.2 Å – there is large scatter.

Why there is such a strange variation of intensities in Fig. S3 between the low pressure patterns below 0.73 GPa, and those above 0.73 GPa?

Similar question can be applied for Fig. S5, but in the opposite – lower pressures seem to have more sample intensity than the higher ones.

12.2 Issues with the CIF file data:

CIF file – the pressure given within the field _*cell_measurement_pressure* should be provided in kPa units (https://www.iucr.org/__data/iucr/cifdic_html/1/cif_core.dic/Icell_measurement_pressure.html)

CIF file – _refine_ls_R_I_factor

If I understand correctly, this value should correspond to R1 factor definition similar to the Ref. (https://www.iucr.org/__data/iucr/cifdic_html/1/cif_core.dic/Irefine_ls_R_I_factor.html).

The values of 2.06456 and 1.63519 provided in CIF should correspond, according to the definition, to R1 of ~207 % and 163 %, which is too large. It should be discussed or explained. It may be a software issue, but it should be addressed.

In the prepared CIF I could not find the number of refined parameters, residual electronic density if the authors indeed used Rietveld refinement which should indicate the quality of the data analysis.

CIF file – **It is a major issue with sigma values for intensity**. Indeed σF() it is much larger than the intensity itself. Here is an indicative and self-explanatory snapshot from the supplementary CIF file showing several fields, including the reported standard deviations of the intensities. These may be related to software processing, but they do not present the data in a favorable light, potentially an oversight.

```
loop_
_refln_index_h
_refln_index_k
_refln_index_l
_refln_F_squared_calc
_refln_F_squared_meas
_refln_F_squared_sigma
_refln_observed_status
   0    0    2     5765.09      5706.97 36337201.01 o
   0    0    3        7.42         7.36     8265.78 o
   0    0    4     5755.20      5911.69  6919154.78 o
   0    0    5       20.60        21.13    18251.82 o
```

Reviewer report

This is the second revision of the manuscript, and I confirm that the authors have made significant progress for the manuscript entitled "The impact of metastability on the high-pressure behavior of cerium". I see that my comments have contributed to improving the manuscript, making the discussion and its presentation, including the derived data (CIF), more robust. I also acknowledge the generally favorable assessments from the other referees. Although the manuscript is now in much better shape than before, I believe that some scientifically important revisions are still necessary prior to acceptance.

Minor, but important comments to be addressed before acceptance:

Abstract:

Line 26:

whereas slow and also moderately faster compression at low temperature stabilizes a pure monoclinic phase

Suggested revision:

whereas compression at low temperature, including slow and also moderately faster regimes (<XXX), stabilizes a pure monoclinic phase ..

At the same time, to make the discussion more quantitative, I would greatly appreciate it if the authors could indicate the maximum strain rate (e.g., <XXX or YYY) to characterize the moderately faster compression. Given the increasing relevance of dynamic compression (e.g. doi: 10.1038/s41563-025-02364-x and other), it is important to provide at least some basis for comparison.

Fig. 1b:

d-spacing 2.5 – 2.6 should be indicated – where 2.5 starts and where 2.6 ends – left of the figure, top, right, bottom? It might be clear for a scientist who analyzed the data, but it won't be clear for a general reader.

Line 148 & Line 175:

coupled with lower levels of deviatoric stresses -> "coupled with potentially lower levels of deviatoric stresses"

are expected to observe low deviatoric stresses only (see SI). We thus suggest it is ..

Supplementary only shows a discussion about the flat and recessed anvils. It is resourceful discussion, but not sufficient. Indeed, many factors should be included, including temperature, Helium (He) is good quasihydrostatic pressure medium when liquid, but is it such at low temperatures when it is solid? What is more quasi hydrostatic pressure medium at low temperatures – liquid He or solid metal Ce?

I suggest that, in a brief surge of enthusiasm, the authors made several attempts to offer a simple, but less scientific explanation of a difficult challenge, with an idea to lead a general reader to a more positive impression.

For an experienced group working under high-pressure it cannot be "*expected to observe low deviatoric*" stress, one may either have experimental evidence for it or may not. Here I suggest an improvement:

It is understood that, under high-pressure and low-temperature conditions, additional undesirable effects may arise, including micro-stains strains (leading to peaks shifts), deviatoric stress (additionally leading to mismatch between the stress on sample material and the pressure sensor); however, given the constraints imposed by the Paris–Edinburgh press and the nature of the sample, we made every effort to achieve the best possible quasi-hydrostatic conditions (see SI for details). We thus suggest that a multiparameter model should be taken into account, including the concept of γ-phase metastability of at this temperature, kinetically trapped through fast cooling.

Line 249-252:

Under low-temperature compression, identically prepared pellets consistently transform into purely monoclinic α ′′-Ce, with no detectable α ′ - Ce. In contrast, room-temperature compression produces a highly textured orthorhombic α ′ -Ce phase with a small coexisting fraction of α ′′ -Ce.

I suggest a revision. This text compares low-temperature compression with ambient-temperature compression conducted by the same group. Under room-temperature compression, a highly textured α′-Ce phase is produced. However, the use of "in contrast" may give the impression that the sample obtained under lower-temperature compression is not highly textured, which may not be the case.

This text contributes to the overall discussion of hydrostaticity of this work and suggests that the authors have to provide additional information how the effects of the texture and preferred orientation were fitted within their full Rietveld refinements. Right now, this important information is missing in the methods section and in the data provided.

Supplementary information:

Table S2

McMachon [10] reference cites none as PTM in SI, but within the actual paper one can clearly read that McMachon et al. also attempted methanol-ethanol PTM studies, with the highlighted statement potentially missed by the authors, although it should contribute to the discussion of "hydrostaticity".

Powder diffraction data were collected on station 9.1 at SRS, Daresbury, using angle-dispersive techniques, with an image-plate detector and an incident wavelength of 0.4447(1) Å [14]. We used Ce from two different ingots of 99.9% and 99.99% purity obtained from Rare Earth Products Ltd, UK; the higher purity sample was double vacuum remelted. Filings from these ingots were prepared and loaded into diamond-anvil cells under dry argon gas without any pressure medium, to minimize oxide and hydride phases. (In fact, test experiments with a methanol-ethanol medium showed no significantly different behavior.) The sample pressure was measured by the ruby fluorescence technique. Structural results were obtained by full Rietveld refinement using the program MPROF [15].

Comments to the replies from the first iteration.

I am glad that my comments helped stimulate work on the CIF file. One should not underestimate its importance, as it will be included in future databases, and it is very positive that the authors took the time and effort to clean it. Not to mention its considerable value as supporting evidence in addressing the reviewers' concerns with respect to the validity of the results.

I cannot fully agree with the statement:

As a result, F^2 values are model-derived and do not have independently meaningful uncertainties, so reporting sigma(F^2) is not standard practice.

But here I would also like to understand the high goodness value of fit (a bit high) in comparison to R-factor values (fairly good and low):

_refine_ls_goodness_of_fit_all 2.49519

_pd_proc_ls_prof_R_factor 0.01639

_pd_proc_ls_prof_wR_factor 0.02064

Since the previous version of the most important piece of evidence – the submitted CIF file had severe issues, I would like the authors to double check and provide an explanation if this value is valid – what it's high value could be related to. The value 2.5 is rather high for conventional experiments, I am not sure if they are typical for experiments related to the Paris-Edinburgh work.

Comments which will potentially affect the stage after acceptance.

Fig. 1b – Nat. Com. policy seem to insist on providing scale for all heatmaps.

Given that the submission does not include raw 1D data, but only figures, I would like to note that, to the best of my understanding, Nature Communications policy strongly encourages if not forces the submission of all raw data underlying the figures in both the main manuscript and the Supplementary Information, either alongside the submission or via online repositories such as zenodo.org.
